# Supplementary material for: Fecal DNA Virome Is Associated with the Development of Colorectal Neoplasia in a Murine Model of Colorectal Cancer
Source: Pathogens. 2022 Apr 11;11(4):457. doi: 10.3390/pathogens11040457 (PMC9025118; doi:10.3390/pathogens11040457)
Supplement: Supplementary file 1 [file pathogens-11-00457-s001.zip › Supplementary Data S6.pdf]

```
>FP_1_4_10_S36_clean_1_215953 flag=1 multi=3.6504 len=7270
ATCATGTTCAGGCGGTACATGGACGGGGAACACATAGCGGATGATGAATCAGTGCTTGGCGTGATCAACTATG
AATTGGCTTTCAGGCGTTCATATATAGCCCATATAAGCAATTTTACACTGATGCTTGATGAATCTCAGTATAT
CCAAAATGAAACGGCTAAACGGTCAAAATTCATACTTTCCTTAAAGCCCGAAAATGTGATCCTTCTATCCGGC
ACACCTACATCCGGGAAATATGAAAAGCTATGGAGCCAGTGCCGGATGCTGGGCTGGAACATAAGCAAGGAAG
CGTTCTGGAATACCTACATTGAAACGGAATGGGTTGAAGATATGGACAGTGGCTATAAAAGACAGAATGTGAT
CGGCTATAAGAATGTGGAACGATTGAAGCGCAAACTGGCGCAGCATGGGGCCGTGTTCATGAAAACAGAAGAA
GCCTTTGAACTTCCTGACCAGCAGGAAATAGCTGTCAGGGTTCCAGTGTCCAAAAATTACAGGCGGTTTATGA
AAAACAGCTATTTGCAGCTGGACGAAGAAACGGAACTTGTGGGCGATAATGTTTTGACAAAGTTCCTGTATGC
CCGCCAGCTGTGCGGCCAGTATTGCCCGGATAAGCTGGAAGCCGTGGCTGACCTGATAGCAAGCACGGATGAC
CGCATTGTGATATTTTACAACTTTACGGCTGAATACCTTGCACTGGTACAGATCACTGACCAGCTGGGGCGGC
CGCAGTCAGTAGTGAACGGCAAACAGAAACAGCTTTTGAACTATGAAAATTGTGGTAATTCCGTTACGTTCGT
ACAGTATCAGGTCGGGGCGATGGGGCTGAATCTCCAGAAAGCCCATATCACTATATATTTTACGCTGCCGTTC
GGAAAGGGAAGCTGTGCCTTATGGGAACAGTCAAAGAAACGGACGCACAGGATCGGGCAAGGTCAGAAATGCC
TATACTACTACCCTTTATGCAAGGGAAGCATTGAAGAAAAGAACCTGATCAACTTGCGGCTGGGGAAGGAATA
CAATGAAAAGTTATTTGAAAAAGAATTTGTTTAGCTTCCTGATCGGCTGCATTATCGGGATCCCCTTTTCGTT
GTACGCCGTAAGCCATAGGGAGCCGGCCACAATCCCGGTGTATTATGATCCGGGGCTGTTTGAACAGTTCAGC
GTGGAACCTGAACACTATGAACCAGCTGAACCAGCTGAACAGGCTGAAACAGTTGAAGCCTATGAAATGGCGC
CGGATGACCTTGAATCTGAATATTACTATGACAGTCTGGAACTGCTGGCGTTATGCGTGGAATCAGAAGCCGG
GAATCAGGGGCTATATGGTAAAAAACTGGTGGCTGACGTGGTGCTGAACCGTGTGGACAGCCCGGACTATCCT
GACAATATCACGGATGTGATCATGCAGCAGAATCAATTCAGCGTAGTGCTGGACGGTAGGATCTGGACGGTGG
AACCGTCAGAAGAAACCTTTGAAGCAATCCGGGAAGAACTGGAACAGCGCACGAACACGGAAATAATTTTCTT
TACGTCAGAAGGATATTCGCCATATGGGGAACCGTGGGGAAAGATAGGGGATCACTATTTCAGTACAGGAAGG
AAGTGAAAAAAAAAGTGGTTATTGATATTTTTAACACTGATAAAAAGTACAACGTGATCTATGCGGATCCGCC
GTGGCGGTATGGTGATCGGAAGTGCAACGGTGCGTGTGAGTTCCACTACAACACAATGACCTTGCAGGAAATA
AAAGATCTGCCTGTAGCCCGGTTATGTGAAAAAGATTGTGTCCTTTTCCTATGGGCTACATATCCAATGTTAA
GCGAAGCCCTACAGGTCATTGAAGCATGGGGTTTCAAGTATAAAAGCATAGGTTTTCAGTGGCTAAAGCTGAA
CCGTTCCGGGAAAGGTTATTTTTTCGGTCTGGGAAGATGGACACGGGGAAATACAGAGCCTTGTCTGTTAGCC
GTCAAAGGAAAACCGTCAAGGCAAGATAACAGCATTTCACAGCTGATTGAATGGCCCATTGGCGCACATTCTG
AAAAGCCGCCTGTCACACGGGAAAAAATCAAGGCTTTGCTGGGGGGGGGGATTGTGTCAGAGGAATAGAACTT
TTTGCCCGGACTACTGCCGAAGGGTGGGATTGCTGGGGCAATGAAGTTTAGGAATGAAAGAAGAAACCGCCAG
AAAAAGGCGCAGGAAAAGGAAGGTGACAAAGAAATGAAAGTTGTGTATGTGTGTTCTCCATACAGGGGAACGC
CGGAAGAAGTAAAGCGTAATTTTGATTATGCGGTGGAACTGACAAAAGAAGTCCTGATGGCCGGCTACTACCC
TATCACGCCGCACTTGTATATCACGGCTTGTCTGGACGATACGAAGCCGGATGAACGTATGCTTGGAACAGGC
GCAGCGTTGGCATTACTGGAAAGGTGTGATGCTGTGATGATTGGCAAGCGTTACGGAATCAGTGAAGGAATGG
CGGCTGAAATCACAAAGGCTACAGATATGGGGATCCCGGTATTCTCCCATTCAGAAAGGACACTGTTCAGGAA
ACTGACGATCCAGTGTGATATGTCAGGCAGCTTTCCAAAGACAACGGTATTTGTGGATAATGAAGCAGTTGAC
CGCATTGAAGAAATTTCTTTTACACATGAAGCTGGCTGGGATCCCATGCTGACTATGAAACGGACACCGAAGG
AAGGGGAAGCAGATGGCACAGGAAAAGAACTTTGAAAACAAGGTCAAGGCTTATTTGAAAGAATCCGGCTGCT
GGTTCCTGAAATACTGGGGCGGTGGCGGTTATACAAAATCCGGGGTTCCTGATCTGCTGGTTTGCTGCAAAGG
GCGGTTCATGGGGGTGGAAACAAAGGCCCCGAACGGCACGCCCACGGATCTACAGATTTACAACTTGCGGAAC
ATTGACAGGGCGGGCGGTCTTGCAGTCCTGCTTTATCCGAAAGATTTTGATATGTTCAAACGTTTGGTAGAGG
ATCCAGACAATAAGAAACTGTATGAAGTTCTGAAATCGAAGTGGAAACACTTTGAAAATATAATCAAAGAAAA
GGAGAATTGAACAATGGCAAAGAAAACTGAAACAACACAGGAACAGGCGGCAAAGCAGACGGAAGAAAAGCCC
GCTGTAAATTATGCTGACATTATCAGAGATTCCCTTATGAAAACAAAGCGTGAGGGCATCGTTGATCTTCTGG
ACTACATGAATGAAATCGGCTTTCTGGAAGCACCTTGTAGCGGTGGAAATCATCTTGCGAAGAAAGGCGGGCT
TGCTGAACATTCCGTGAACGTCCTGACCATAGCGGAAAAAATTGGTGTTGCGCTGCTGGGCGGCGCAAGATAC
AACGAAGTTCAGGACAGCGTTGTTATTGCCGCCCTGCTTCACGATCTGGGGAAATGCGGCGATTATGAAAAGC
CTATGTATGTTGAAAGCATCCTGAAATCTGGGAAGCAGTCTGACGCAAAGCCCTACAAGCGGAATCCTGAACT
GTCTGCCGTTCCCCACGCTGTACGGTCTATCAAGCTGGCCACGCTGTTCATTGACCTGACGGAAGCCGAAGAA
TGGGCGATCCTTTGCCACGATGGGCTGTACGATTTTATGAAGTATGACCTGAAAGGTAAAGAAACATGGTTAC
AGATGATCATTCACTGGGCTGATATGTGGGCCAGCCACATTATTGAAGGGGGTACAGATGAATAATGATGGGA
ACTATGTTCTGTCAGTATGAAACACCATGTGGCTATTGTGTGCGACTTGACAAAGAATGTCCTGAAAAGGGTA
AATGTGCGCCACGGAAAAAGGCAGCCGCAGCGGTAGCAAGTCCTGAATCTGATGAAAAGTTATTAAATCCGGC
TGGTGAATATGCTGTTAAGTTTGCAGAAAATCATGGAATCAGCGTTGATGAAGCCATGCGGGAACCGATGGTA
AAAGCCCGGTTTGCAGCATATGCGGCCGGTGGAATTTAAGAAAGTGAGGTATGACAAGATGGAGCGTGATTAT
TTAGAATGGATCAACCGGACATATAGAAAGCTGGTTGACGATCAGGAAATCCCTAAAATGGAATTGAAAACGC
CGGACGGCGGCAAAATTTCCGTTTACAGGGTGGGTGATGTTATCCGCTGTGACATTAAGCGGGCAGAAACTAA
```

AAGAAAGTGAGGTATGACAAGATGGCACAGAAAATTTTAATCATGGGGGACTCAGGAACCGGGAAAAGCACCA
GTTTAAGGAACCTTGATCCCGAAATCACGGCGGTGGTCAATCCCGTGGGGAAACCGTTACCGTTCAAGTCCAA
CAATGGGAAGTTCACCATGCTGAACAATGAAACGAAGTCCAGCAATATCATGGCATGGATGAAAGGTCAGGCG
AAAGCCGGGAAGAAGATCCTGATCGTGGATGACTTCCAGTATTTGCTTTCCATTCCCTATATGAACCGCATAC
ATGAAGGGGGCTGGGATAAATGGAATGATTTTGGTGACGATTATTTCAAGCTGATTGACATTTGTGCTGACCT
TCCGGCTGACGTGCGTGTGTATTATCTCTCTCATTGTGAAACGCTGGAAAATGGGATCACTACCATTAAGCTG
ATCGGAAAGCTGTTAAGGGAGAAAATCACCATTGAAGGGCTGTTCACTATCGTTTTGAGAACGTCCGTTATTG
ACCAGAAGTATTTCTTCCTGACGCAGAACAGCGGCAAAGATACCGTAAAAAGCCCTATGGGTATGTTTTCAGA
ATATGCGGTTGAAAATGACCTTGCCTATATTGATGATAAGGTATGTAACTATTATGAGATAGGCAACTACAAG
AGTGATGCAGAAATGGCGGCACGGGATCAGGAAGTGGCCGGCGGTATTGAAAAGCCGGATCCGAAAGGACGCA
GGGCAAGGGGTACATCCGCAGCACAGAAGGGCGAACGTAAGACACGGGCGCAGGTGGAAGCTGAAAACAATGA
AAAGATCGGTCAGTAGATGGAACAGGTTGACAAGGCTGTTTCGGAAGCTGCCGGGGATCGTGAAGAAATTCCG
TTTGAAGAAGCCGCTGCTGCCGCCGATACCGTACCGAAGCCGGATCTGGAAAAGCCGCCCCGCCGTACACGCC
GGGAACGTCAGGAAGAAGCTGATCAGAACAAAGCTACAGAAGATATGTATTTTTACCATGCGGAATCAGATAG
TTTCTGGATGCTGAAAAAGGGTGAAGTATTCCCTAACAATATTGATGCACAGTGTTCTGTTCAGGTTACAAAA
GAAGAATATGAAGCAAAACGCTGTAATATCCCGGATGGACAGGACGAAGAACCGCTTCCCATGAATCCCCCTG
TAGAGGGTGACAGAAAGCCCCGTGAACGCAAGACAAGGGATATTCAGCCGGAAGGTCAGGCAGAACAGCAGGA
AACGCCGCAGGAAGCCGTTCAGGAGCCTGAACAGGGTGGATCTGCAAGGTCACGCCGTACAAGGCGCACAAGG
GGGTAAATGTGGAGCAGAAAGTAAAATGTGATAAATGCGGAATGAAGATCTTTATCAAGCTGAAAAAGCAACA
GAAGGGCGATCTGGAATACCAGTATTTCACCTGTAAGCACTGTAACACTTCTTATGTGGTATCGGCAACGGAT
CCGCCCTTGCGCCGGGAAATGCAGAGGTATGAAGATCTTGTGAAACAGGCTAACGCACTGGCGCAACAGGCGC
AGGAACTTTTACAGATAAATGTGAAGCGGTGCAGGGAAATCATGGATCAGCATCCGCTTGAACTGTAGGTAAA
AAATTTTAATTTTATGGCCCCATGAAGGGCAGAAAGGATGTTATAATTATGGCAGTAGATTTTTCAGCATTAG
ACAAGGCAGTTGATCAGGAACAGTTAAGGAAGGACGTTGAAGAAGCGAAAAACAACAGCGGCGATATTCCCAA
AGGAACCTATATTGTGGGGATCGACAAGATGGAGATCCGGGCCACGAAGGATGGCCGGCCTATGTTCTTCATT
CAGTGCCGTGTCAAAGAAGGGGAATACAAGAATAAGTGTGTATTTATGAACCGGGTTATTTACGGGACAAAGA
ATGACGGCAGCATGATCCAGTCTGTTATTACCCTGCTTGACAAGCTGGAAACGGAAACCATCCCGGAGTTCAA
CGGGTATCAGGATTTTGTGGATGTGGTGGCTGACATTTACGAAGAAATTCAGGGCAAGGTTGAATGTGAAATT
GACTATGACAAGGACGCTTTCAACAGTGTCAGCATAAAGGAAGTATTTGACGTATAAATTTTTTTACCCTAAA
AGTTAAGACTACTTAACTTCAAATGAAATCAGAGGGCCGCTGTTAAAAGATTCTTAACAGCGGCCTGATGGAC
AAAGAAAGGGTGATCAAGATTTTATTTTATGACTTTGAAGTTTTCAAATATGACTGGCTGGTTGTGGTGATTG
ACAGTGACACGCACAAGGAACACGTCATAATAAATGACCGTGACCAGTTAGAACGGCTATATGAAGCAAATAA
ATACAACATATGGGTGGGGTACAATTCCCGCCACTATGATCAATATATCCTGAAAGGGATCCTTTGTGGTTTT
GATCCAAAAGAAATAAATGATTTTATTATTGTTCAGCATATGGACGGCTGGCAGTTTTCTGACCAGCTGCGGA
AGATCCCCTTGAATAACTATGACGTGATGCCGAACCCGCCCGTAGGATTGAAAACACTGGAAGGGTTTTTAGG
ATCCGACATAAGGGAAACGGAAGTTCCTTTTGACATAAACCGGAAGCTGACACGGAAAGAAATTGAACAGACC
GTGTTCTATTGCCGCCATGACGTAGAGGAAACCATAAAAGTGTTCATTGAAAAGATAGATGATTTTCATGCTA
TGTATGGGATTGTCAAGGCTTTCCCGGACATGGTGAACTTGTCAAATATAGGCGATACGGAAGCCCGGATCAC
TTCTAAAGTGCTGGGGTGTGTCAGGAAGAATTTCAGGGATGAATTTGATTACTTCTTTCTGCCCTGCATACAG
CTGAAAAAATATAAAGCGGTTCAGGACTGGTTTGAAGAAAAGAAACGGGAAGCCCTGTCAAACAATATTCAGG
GGAAAAACGATGCCGCAAAAAAGGCATGGTATAAAGCGCAATCTTTTGAAATGATTGTAGCCGGTGTTCCGCA
TGTTTTTGGTTTTGGTGGGCTGCACGGTGCGCCTGATAAGCCTATACACAAGAAGGGCCTATTGCTTCATGTT
GATGTAGGCAGCTACTACCCTTCTTTACTGCTGGCGTGGGAAC
>MA_1_1_23_S45_clean_1_24213 flag=1 multi=4.0000 len=6438
GCGTATTTAATTGCAGATGTTTTCCAACGCCAATTTTTATCGAGAAAGACGATGAAGAAAAAATTTAGAGACG
GAAAATTCTTCACAACCGAAAAGATAAGCCCTCAGAAAGAAAAAACACCTGAGGGCTATTTGTTGTGTAAGTC
AGTACCGATTAGCCGTATCGGAACATTTGAATATTCCGGAGTGGAAGCTGGTTTGTCAAACGTTTCATCAGTG
ATGTTATCCAGAAAAGAAGATGAATTGTTTAACTCTAAAACAGTGACCTCTTTTGAAGGGAAACCGGTTGTAA
TCAGTCACGCCCGCTTCGCCGATCCTGACAATTGGAAAGACATTGCTGTCGGGACAGTTCAAAACGTGCGCCG
TGGAGACGGAGAAAACAGCAGTTTCCTTTTAGCCGATCTACTGCTGACTGACAGAAATGCAATTGACGCTGTG
GAATCCGGACAGCTCAAAGAGGTCTCTTGCGGTTATGACGCAGATACAGAAGAAACCGATGACGGAATTAATC
AAGTTGGAATTGTGGGAAACCACGTTGCTTTGGTAGTTAATGCCCGGTGTTCGGGCTGCAGAATAGGAGATGG
AAATATGAAGCCAAGCATTAAAAATCGTTTGCGGAAATTTTTCCGTGACGGCGATGAAGATGGATTCAACGAA
GAGTTGGACGGCATGACGTTAGCCGATTCAGAGGCTCAGCCGCCTCAGGAGCCGACCCCGCCGGCTGCTCCCT
CTTTGGAAGAAAGATTGACCAAACTGGAGGTAGCAGTCAATGCTCTCTTAGAGGCCCAACAGGCTAAAACAGC
TGATGCAGAAGAGACTGCCGCAGAAGCCGATGTCACCAACTCTGTTGACAACGAAGATGAAATTGTTGAGGAC
GAAACAGCCAAACAGGTGCTTGCTGACGCTGAAACATTGTGCCCGGGCATGAAAAAACCCGTAGGCGACTCAA
AGGACGGCAAATTCTCCAGAGGCATGATTGAAAGGGTCATGCGCACCGCTCTCAAAGGCTCAGGCTGTAAACA

GTTCGGAGATTCTGCGGAACTCGAAGGCAAAGCGCTGGACATTGCTTTTAAAGCCGCCCTTGAACTAAACAAA
GCAGGAAAGAATCCTAAAGCTCGTAGCTTCGGCGACAGCTCGGAAACGCCTGAAGTCAACTCAATTGAATCAA
TTCAGAAACTTTTTAACGACTATTGGGAGAAAAAATAATGTCTTCTTCTTTTATTGGTACATCAATGTCCCGT
GGTTTTGCCGGTGAAATTACACGAGGAACTTTCGACTACACCACAGAGGTCAAAGAAAACGATAGCACAACTC
CGGTTGGTGCTCCCGGCTTGCTTGTTTCTTTGACTTCGGCCGGAAAGGCAACAGTAGCAACTGACGCCACAAA
AGTTTATGGCTTTTCAGTGCGTGATTACCGCCAAGTAGGCCCTGACGGAAAAGTCTGGCCTGCAGATCGTTTT
GTTTTAATCCTGCGTAGAGGCTACATCGCTGTCCTTGCTGACGGCACCCCTGCTGTCGGTGGCGAAGTCTACA
TCGGGACAAACGGAAAAGTCACCGCTACGAAAGGCGCTGGTGCTGTAGCAATCCCGAATTGCCAATTTATGGG
CACTAAGGATGCGGATGGCATTGTTGAAATCGCATTTAACATTTAAAGGAATGAAAAATGAGCTTGAAATTTG
TTGACTCTGAATCAATCTCTTCCACAGGTGCATTTCTGGTCGGTGAACTGGAACGCCTGGATGCAAAAATCTA
TGAGCCGATTGTCGATTTCACTTACGGTCGTGACATTGATTTAAGAGAGGATGTCACCATCGCCGATGAAGTC
TCCTCCTTCCTGCTCTCTGACTACAAAGGAGGATTCGGCGGCACATCCGTAGGAAAGAAGTCCTTCATTAAGG
GTGCTGACTCTACTCCGGCACGTGTTAGCGTTGGCATGAAAAAGATCGCCACTCCGTTGACCCTTTGGGGTAT
GGAAGTGTCCTACACCATCTTCGAATTAAAGAAAGCCATGCAGGCAGGCCGTCCGATTGACGCACAGAAACAC
TCTGCAATGCGCTTGAAGCACCAGCTAGACATTGACACTCAGGTCTATATCGGTGATACCGAAGTTGGAGTTA
AGGGCCTTTTGAACTCTGATCAAGTCACTAGCGAAAACGTTGGAACTTGGACAGATTCAACTGATGTTAAGAC
AGTTATCGGCTACTTCAATAACATCCTTGAGAAGGCATGGAAGGCCACCGAATACAACAGAATTCCTCGCAAT
CTGCTTGTACCACCGGCAATTTTCGGCAAATTAGTAAGCACTCAGCTTCCTAACACCAAGATGAACCTTTTGA
GATACGTTGAAACAAACAACCTCTCTACTGCAAACGGTGGTACTTTAACCATCCGTCCGGTTCGTTGGCTGGC
AGACAACACTCTGTTTAGTGCTCCTCGCATCGTGGCTTACACCAAAGATGTCGATGCTGTGCGCTTCCCGCTC
GTTCCGATCGCTTCTTTACCGGTTCAGTACCGCAACTACGAACAGGCGGTGCCTTATTATGCGGCTCTTGGCG
GAGTTGAGTTCGTTCGTCCGGAAATGGTGTACTACGCAGATTTAGCCGCTGCCTCTAGTTAAGGAAAAGGGGG
CTGAGATGAAAAAAATCACTGTAATCAGTCCCGTGATTCTCAACACATCCGGGGCGAGCTATCAGTTCGCTCC
GAAAGTGGAGTATGAGGTAGCTGACGACATTGCGAATCATCCCTATCTGCGTGGTTTTATTTCCACATGTATA
GACATTTCAAAACCCAAAGAGGAGCCGGCTCCCAAGCGGGCCCCCTCAACCAGACGGGGAGTAAAAAATGGAC
GTACTAACCATTGAGCAATTTCGGGCGAATTTCCCCGAATTCATTGAGGACAATTTCCCTGATTCAGCGGTCA
AAATTCGTTTGAGTTTGGCCGCTAAATTTTTCTCAGAGGAAACATGGGCAGACCCCGAAATTAGAAACCATGT
GATCGGCCTGTATGCAGCTCATTTTCTGAAGCTTGAGGGTTCGGCAGCAGAGGGTGGCCATGGCGGAGACAAC
TCAGCTCTTGCACAAGTGTCCTCTATGTCTGTTGACGGTGCCAGTGTCAGCTATGACACCAACACTTCCGCAG
AAGATGGTGCGGGAACTTGGAATTTGACCGCATACGGCAGAGAGCTGTGGCAGTTAATGCAGTTGTTCGGTGC
AGGAGCCAGACAGTTATGATGAAATCAATTAACGTGTCTATCGTGCGCCACGACAAGGAGTTAAACGCTGCCT
TGAAGAGGCTTCAAAAAACCGCTATCTACGTTGGCATCGCCGAGGGCTCAAAAAAGGATAAGCGTCAGGACAA
AGCCCCTGACAATCACTTGCTAGGCTTTGTTCATGAAAACGGTTCTCCGGCAAATAACATCCCTCCTCGTCCT
TTTCTCGTTCCGGGGATTGAAAAATCATCTCAGATGATTATCTCCGGATTAAAACGTGCGATGAACGCTGCCC
TTAATGGGGATGAGCAAAAATGCTCTGAGGCACTGGAGCGTACGGCTATTCAGTCTGCATCGGCTGTTAAAGA
GTACATGCAGACAGCTGAATTCGCTCCTCTCAAACCTCAGACAATTGCGCACCGCAACCGCTCCAGAATGACA
GCAGGCAAGCGCAAAAACGAGAACCTCGGAATCAACATAAAACCGTTAATTAACACGGGCCAGTTACGTAACG
CTATCGATGGCGTGGTGGTCGTGGAGGAATAAATGGCGACATTAGATGTAGAACGGGTTATCCGTTCACCTTT
TTTTACTTCACCCTGCAAGCTCGTAGCGGTAAAGGAGTCTGCGGACGCCTACGGAAATCCGGTATGGACTGAA
ACGGCTTCGGCTGAAGTTCAGGCTGTAGTCACCTCCGATTTGCGAACCATGCAGAGAATTCCTGAAGCTCTCC
GGCACACAGGTTCGATTTTAGTCAGAGTCATGATTGCCGATATTCCTGAAGGCTTTACCGGCACAGGTTTTGA
TGAGATTCATTGGAGAGGCCGGCGTTTTACTGTAACCGACTGCGCAGATTATTCTCAATTCGGAAAAGGATTT
TTGCGCATGATTTGCGCCCCTATGGAGGTCGACAAATGACCGTGTTTGATGACAGCAGAGGAGCAAATGTTTT
GCGACCAACATCTTCGGATAACGAATCGAACTTTCTTGATCCGTTGAGGATTTGGGTCTCAAAAATTTTAGGA
TTCCCTCCTGAAATTGTCCGAGCCAGCTGGCTACAAAAACCCGGAACTCAACCAAGCATTAACAAAAATTGGT
GCGCCCTAGCCGTTAAAGGAGCTCAGAGCACTCCGATTTATCAATTCGGCAAGAAAGGCCGTGTTGACGATCC
CGAAAGCGGTGACACAACTCAGGTATCTCACGATGACTATCAAGTTGTTATCAGTTTTTATGGACCGTCTGCA
ATGTTTAACGCTATGAGGTTTCGGGACGGAGCACAGCTGCTGCAAAACAGAAGAAATCTGGAGAGTTACGGCC
TAACCCTCATCAGACTCGACCCGCAGACATTGAGGCTTCCTGATTTTGCAGGAGAACAGTGGATCGACAGATA
TGACGTGGAATTCCATGTCGGCAGAAAAGTTTCAAGAACTTATGGAGTCCGCACTGTTGTGAGTGCTGATTTC
GACATTTTTTTTAGAAAGAGGTAAAGTATGAGCGTATCACCTAGGCTTCCTGTCTCAGAGGTTGTAAACGTCA
CAATCGAGATGGCACCGGTAGCAGCCGCACTCCGAAATTTCGGAGCGATGCTGGTTTTAGGAACGAGCGATGT
TATTGACGTTGACGAACGTATCCGCACCTATTCAGGTGTTGAAGCTATTGCCGCCGATTTCGGTACGAACTCG
CCGGAATATTCGGCTGCAGTTACGTTTTTCGGTCAAAGTCCTCACCCTTCCCTGCTTAATGTTGGGCGTTGGG
CAAAAACGGCAACATCCGGACTTCTCAGAGGGAGAATGTTAGCTGTCAGTGAACAGCAGATTAGCGACTTTGA
AAAAATCACAGCCGGCGGTTTCACTCTCAAAATTGACGGCGCCTCCGTAACACTTGAATCTGTTGATTTGAGT
GCAGAAAGCAATTTGAACGGCGTGGCTACTCAGATCACTAATGCTTTATCTTCTAAGGGCACTTGCCTCTGGG
ACGGAACTAGATTTGTCATTAAATCGGCCTCAACTGGAACAACCTCATCGGTCTCTGACGTATCAAAAACCGA

TTTATCTACCTTGTTGGGTTTAGATGAAGGAACTTCGAGGGTATCCGGTGCACCTGCAGAATCATTAGTTGAC
GCTGTAACAGATTGCCTTGACTTTACAAACTGGTACGGCCTTTTTGTTTGCTCCGGATGGACAGAAGAAGATG
CATTGAGTGTTTCCGAACTGATTAATGCAGCCAGCCCGTCACGAATCGTTGCATGGACATCTGAAGATACCGG
CGAGATGGATTCAACTGATTCCACTTCCCTCGGCGCAAAATTAAAAGCTCGTGGCTACAACCGCACCTTCGAA
GTATTTTCAAGCACAAGCGACACTGCTTCTGCATCCGTTTTAGGACGCATGAGCACTATCAATTTCGAAGGCT
CAAATACAACTCTGACACTGAAATTTAAACAGTTGCCGGGAGTGCCTGCAGAAAATCTCAGAACCTCCCAGTC
GTTAGCGTTGCGCTCTAAAAACGTGAATGTTTTTGCCGCATTCCAAAACGATACTTCGATTTTTAAAGAAGGC
GTGATGTCCGGAGGCTGGTTTATTGATGAAACCCACGGCCTTGATTGGATGCAGAACAGAGTTGAGACAGATT
TGTGGAATTTGCTGTACACGAGCAATACAAAAATCGGTCAGGACGAAACAGGTATGACCGCGATTCTAGCGAC
AATAAACAAATCGCTGGATGCAGGCGTCAGAAACGGATTAATTGCTCCGGGAGTTTGGAACGGTGACTCCTTC
GGTTCACTTGAAAAAGGAGACACTCTGACAACCGGCTACTACGTTTATATCCAGCCGTTGGAAGAACAAGCGC
AGAGCGACAGAGAAGCCCGCAAAGCACCGCCGATCAAGATTGCTATCAAGCTTAAGGGAGCAGTTCATTTCAT
CGACTGCACTCTCACAATTAACCGCTAGGAGAAATTTAAATGGCAACTTATTCTTTTATGGATGTAACCGCCA
CTCTGGCGGGACCTACAGGTTTTATTGATCTGGGTTATGGTGCTGCTAATTCAAAAGAGGGAATTTCCTTTGA
GTACAGTCAGCCTCGCAACAACAGAACTGTGGGGGCTGACGGTGAAGTCATGCACTCTCTGAGAGCAGATAAA
AGCGGTTCAATTACCGTACGTCTTTTGTATACATCACCGGTTAATGCAAAACTCAAAATCATGTTTAACGCCC
AGTCTCTGAGTTCA
>MA_1_4_14_S91_clean_1_272093 flag=1 multi=4.0000 len=5607
TTCGGTCAACTCGTCGTCAGTCAACCGCTCCAACGTCTCAAAGAGATAATTGTTGAGGTCAGTCAGAGTGTTC
CGCATCACGCATCCCTCCGGCTGAGCAGCTGGTGGGTGCCGTCCCGCATAGCCTTTTCTTCGTCGGACATCTC
ATAGCCCAGGGAGACCAGGAAGTTGTAGAGACGGTCAAGCTCACTGTTCGGCTCATGCACGATCTCGTACTCC
CGCTTGTCGGTGTTCCACCTCTCGTCCCAGTAGCCGTTATCATCCGCATCAACAGAGGCATAGGCGCAGACCA
GCAGGGCGTACTCCGGGTGCTCGTCGGCGCACTCTTCCACCATGGCCGTGAAAGCGGGGTAGTCCGTGTTGTC
GTCAATGCCCAAATCCAGGAGTTCCCCAGTGAGCTCAGCATTGATTTCATCCCGGCCATATTCGCCGTCTCCG
ACGATAGCACCGACCGCATACTTGCAAATCTCAACCAAGTGCTTTTTGGCGGCGGAGAAATCAGAAACGAACT
CAACCCGCAGGGAGTAGTGCCGCTCGGTGATTTCCCTCAGCTCGGCCTTGACGCGGTTCCTGGCCCGGTCGTT
CTCTTTGCGCTGGCGCTCCTCCTCGGTTTCGACCCGCTCCTGCCGGTCTTTGTAGAGGTCGATTCCGTTGGTG
CCAACGAGGTAGTAGTATCTGACCTCGCCGGCGTCCTCGGGCCTTTCCACCATCTTGTCTTTGGGCGACCAAA
TGCCGTAGTTGGTGACATAATCCATGGGAACCGCAGTAGAGCCAACGCAGTCCCGCTTTTCGATTTTGAGGGC
AAACGCCTCCAAGTCGGTTTCCCACTGGGCCATCCGGCGCAGGTTTTTCTCCTCCTCAATAGCCTTTTTCAGA
ACGTCCCTGAAATTGGCGGTGCCGATGGCATCCAGGGCCTTGTTCTTCAGCTCGGGCGTCTCGATCTTCTCCA
GCTGCGCATAGTCCTGGAGGGTGGCACCCCGGGCCTCGGACTTCTTGAACTTGTCCGCATCCAGCTCCAGGAG
CCGCACCCGGCGGCGCACGGTGCTTTGGGAGAAACCGCAGTCCTTGGCGATACTCTCCACCGTCTCGCCCATA
TCCAGCATCATCTGGAAGCCCTGGGCCTGTTCGTACACGGTCAGGTCGCTCCGCTGGATATTCTCCATCAGCA
TGGTTCTGACCTGCTCCTGCGGGGTCATTTCCATGGCCACGCAGGGAACCACCGACAGGCCGGCCTTCTTGGC
CGCTGCCAGACGCCGGTGCCCGATCACGACGGTGTACTGGCCGTTGCTCATGGGCACGACGGTGAGGTTTTGC
AGTACGCCGTTGGCCTTGATGCTGTCGGACAACTCCTGGATGTCCCCCAGGTTCTTCCGGGGGTTGTCCGGGT
GCGGATAGATCTTCGAGACTTCGATATATTGCAATTCAGGCATGATTGTCTCCTTTCAGTTGTCAAAAAAGTG
TCAGTTGTCCCGCCGGGGTTTCCCCCAGGGCCTGGGGCACGGGTTCCGGTGGCTGCTCTGCCGGCGGTTCTTC
CTTGGGCACCGCCTCTCCGTCGGCTGGGGGCTCGACCGGCCGTGGGCCGGAACAAATCATCCGGTCAACAGTC
CACCACAGGCGGCGGTAGTGCCACACGTCCCGGAAATACATGGGGGTGTACCAGATGTTCTCCCCGGGAGTGG
GGATTAGCGGGTTCCCGTCGATGCCAGTACACGGATGGAGGATGCTGTCGCCAATCACCACATACCCAGGGCA
CCCCAGCAGGCTCAGCTGGATATAGCACATACACCCCACGATGTAGTCAATATCCTGGGCCACGAACAGCACC
GACGTCTGATAGTTCACGTTTTGGAGCTTGCATTCGTTGGCAAAGGCCACCAGGAGGGCCCCGGCGCCGCAGG
CTGGGTCGTTGACGGACACCCACCCCCGCCGCTCCATCTTCTCCTCCAGGTCTTTGCCGTAGGTCATTTTTGC
CATCATCTTGCACACGTTGTACGGCGTGAAGAACTGGCCGTTGTGGTCGTTTCCGAGATCCAGCGCCATGAAC
ATTTCCCCGAGGAAGTCCTGGTCTGGATTTTCGTCGATGCCGCTGACTACCTCAGCGAACATCCTGCAGATGC
ACTCCAACTCCTGCTTGTCGTACTTGGAGGCCAGGGACATATAGGTCTCCTCCCTGCCCTTGGCGTTGCTCTT
GTCCACGGTGTTGGAAATGCTGATGGCAGCCATGACCACAAAATCTGCCCAGACAGTCCAGCGATTGTGCCGC
CTACACGCTTGGTCGAAAATGCGGATAAACTCTTTTTGCTGCTCATTCCGCAGCTGCCGGGTGACATTCCGGG
CCATTACGACCCCTCCCAGTAGTAGGGGTAGCAGAATACATGGCCTCCGATGGCGCCCCACACGTTGTCGTTC
TCCCCCTTCTGGCTGAAGAACACAACCTCCAGGGGCAGGATGCTCGGCCCGCTCAGGGCGGTGCTGATGGCTA
TGTACTGCGCCTCCGTCGGCTCGGCGGACTGGACGCTCTCGGCGGTGGAGAACTGCCCCTCCTGGTAGATCAC
TTCATCCAGCGTGTCGGGGAACGCCGGGGACAGCATACGGTTGAACACGACCTCGGCCACGGCCTGCTGTCCC
TCGAACGGCTCCCCCCTGGCCTCCAGCCAAATTACCCTGGCCAGGGTGTCCGTCTCTGCGCCGGTCAGCTGGA
TTGCCCCGTACCTCCCCGGGCTCTCCGACGGTACGGCCTCTGGCGTGGCCTCTGTCGCCTGTTCCTCCGGGGG
GATGGTGGTATAGGGGGCGTCGATAAACTCGTCTGTCCTCGGCCCCTGGGGGCCCCTGGCGATACACAACGGC
ACCGCCAGGGCGAGGAGCAGTACGAGAACGAAAAGGAATGCTGCTTTGTTCATCGCTAAAAAGCTCCTTTGTT

TTCGTCGCGGTCAAAGTCATAATCCTCGGCTTGGCCGAGGGAAGATATGCCGCGCTGTGCGAGGCGTCCGAGG
ACGCCGTTGATGTACTTCCAGTCCCCTTGTTTTCCGGCAAGGGCCGCTTGCTCAAAGGCATACATCAGCAGGT
CGATCCGGTCTTTGGGGAAAGTTACTGTCCACGTCCCGGTTTGCTCGTCGCGTTCGCTCAGGTGGATGGCCTG
GAACACGCTGGCCCGGTCTGCGTCGGTAGGGGGGCGTCTGGCAAACCTGGAAAAGATGGCGTCGGTATAGGCG
GCGACGGTGACAAGCTCCTGTTCGGAGACGCCGAAGTAGGTGCTGGCATCCAGACAGCGGTAGCCCAGGTACT
CGGTCACGGCGCTCTCCGCCGCCTCGCGCGCACGCGCACCACCACCAGAGAGAGAATTATTATCCTCTCCTAA
CCTAACCTGACCTAACCTATCCTTACCTAACCTATCCTGTGTATCCAGTATGGATACATCGGCGCAAACCGCT
GGAATTGCTGGTTTTTCCTCTGCTGTATCCTCCTTGGATACAGCTTGGATACACGGGCGCGCGCCCTCGCTCG
GATGGTCGGTATAGGCTCCGTCCGGCTTGAGGTATAGGGTCGGCTTGTAGCGGTCTTTCTGGATATAGTTTGA
GATTTTCCAGTGCTTCAGCACAATGACGCCGCTATCAAACGTGAGAACAAATCGTTTCAGGATCAGCAGTTTC
AAATCGTCGTCCGCCGCCCCAACCAGCTTTTGTATCCGCCGGGCATTACTCACAAAACCGTCGTCGTCCGCCC
TCATGCCAAGGTGGAAGTATAGCGCCTGGGCGGACAACGGCATATCCATAAAGGCATCAGTATCAACGATTTG
CAGGGAAAACATTCTGCGCTGAGCCATACTCTACCACCTCCGCTCAGAACGGGAGTTCCCCGTCGTCCTCTTC
CAGCTCGTGGAACTGGTTGTCGCCGTAGCCCCCGGCGCTATATCCCCCCGGGGCGTAACCGCCATATCCGGCG
TCGCCATAGCCGCCAGCGGGCGGATACTGCTCAGCAGGCGGCGGGCCGGAGGACTCTTTCTTGCTGTCGCCGA
AGTAGCAGCTGTCGGCAACTACCTCAATCGAGATTCGCCGGTTGCCGTCCCGATCCTCCCACTTCCGGCTCTG
GAGCCTGCCGTCCACGACCATCATCCGGCCCTTGGTGAAGTAGCGGCTGACAAACTCGGCGGTATTCCGCCAC
GCCACAACGTCGATGAAGTCCGTGACTTTCTCTCCGTCCTGGCCCTTGAAGTCGCGCTCCACAGCCAGCGAAA
AGGACGTGACGGCGTTGCCGGACTGGGTGTGCCGCAACTCCGGGTCACGGGTGAGACGGCCCATGATAGTGAC
ACTGTTCAGCATTGGCGGCTCCTTTCCTCTTGGCAGATCTGAACGACCCGTTCACACCGCGCCGTGTCAAACA
TACCGATGTGGGTTTTCTCCTGGGGCAAGCCCATCTGCTCGGACAGCCAAGCATAGGCGGCCTTGCGGTGCCC
CTTAAAGCGCCCCTCTTTCCAGAGAGGGTCAAAGGCGGCGTGAGCCTGCTTTTTCAGTTCCCGCAACTCCATA
TCAGCCAGCCGCCCTAGGGGACGGTCGGTGCCCTTGTGTACGCCGACATAGGCATACCCAGGGCAACAGCGGC
AAAGGTAAATCATGCCGTAGCTTTTGCCATAGATAACCTTGCTGTCCACGAACTCGGCCCGTCTGCCGCAGTA
GTCGCAGTAGACTTCTTTCACGGTTGCCATTCCTCCTTGTATCTGGCAATTTGCTCCGGGGTGTCGGTATCAA
TACCGAGGCCCTTTGCAACATAGATAGCGCCGTCAATCAGGCGGGCCATCTCCTTTGTGTCCAGGGTGTGCGT
ACGCTTGTAGAACAGGTAGCAGTTATACTCCCGCCCCTCCAGCGTCATGGTCTTGTAGAGCCGGTAGTAGGGA
TAGAAGTCGTCCATATCGGCGGACACGGGGAGCATGGCCCCCAGGGTGTTGCCGTCCTCGTCTCTGGCAAGGG
CCCCGTACTCCACCACCAGACTGCGCTTTACCTCGTCGTCGCCGAGGCTCTGGGCCTCGGCGATTTTGTTGAC
GAGGACATGGAAATATGCGTTGGCGTCTCTGCTCCGGGGCTTGCGCCACTTCTTGATCTCCACGTTGATGTCG
GTGTCTTTGAGAGCGTCGTAGCGCTCCCGGAAGTCGCTGGTCACGGTGATGGTGATATGCTGCTCTCCATGAG
CGCCAAAGGTCAGGTCTTTCAACCGGCCTTTCATTTCGCCAGCCACTTCTCCTGGTAGAGCCCCAGGAGATCA
CGGGCTGTCAGCCAGTCCAGGAAGTCGGAAATGACAGGGAAAATGCTGGGGGTCTCCTCGCGCCGGTATTTCT
CCTGCCAGACGTCGGTTCCGTTGCTTACCAAGTAGGTGAAGTGTTCCGCCTCCGGCACCAGCTCCAGATAGGT
GGGGTGCTGGGTGCTGTCGAAGTATTTGCCGCGGTCATAGCCCTTGGAGAACTTGATGTCATAGATGCCCCCA
GCGAGAAGCGCATCCAGCCGGCCGTAGAGAACGAGCGTCAGGCCGCCCACGCAAACCTCTTTCCGGGCCCGGT
ATTGCAGGACGCCCCCGCCGACGATGGCGGCGATCTTGGAGGCGGCGCCGTACCAGCGG
>MA_1_4_20_S31_clean_1_93789 flag=0 multi=5.9877 len=5020
AGCGCAAGGAATTTATGCGCCAGAAACAGCAAATCGTAAATACTCTTTTCCTGCAAGGAAATAACTGATTCTG
TTACCCCCCCGCCGAAAAACTACAAAATTTCTTCGGCGTTTTCTTACAATTTCAAATTGGCGTTGACAGTACA
TTCTGGAGAAAATCAGGGTCGAGGGCGTGCTGCAGGACATCCTGGCGAAGAGCAATGGCGAAAACGTCACCGT
CACCTACGGCGGCAGGGAGCAGACTCTGACCGCAGCACTGGCGGCCATCTTCGCCAGTGTTACCGAGCTGCCT
ACGGCGGAGGGTGTGGTTACCAAAATCAGTGCTGCTATCAGTGAACTGATCGGCGGGGCTCCGGAGACCTATG
ACACACTCAAGGAGATCGCGGATTACATTGCAGCCCACAAGGACGTTTCCGACGCCCTCACCGAAGCCATCGG
CAGGAAGGTGGACAAGGTGGAGGGCAAGGGCCTGAGTACCGAGGACTTCACCACAGCGCTGAAGGAGAAGCTG
GAGGGTCTGCCGGAGAGCATCGCGGAGGCTACCACGGAGAAGGCCGGTCTCCTGAGCGCGGCGGACAAGGCCC
GGCTGGACGGCATCCGGGGCGTCCGGTATGGCACGGAGGCCCCTGCGGACATGAAGGATGGAGAACTCTTCGT
CCGGGTCATCAGTGTGGCGGAAGAGACCTGAGAAAATGGTGCGGCCCGGCAGCCATAATGCAACTGCCGGGCC
GCCCGGGGCCTGATGACTAAGAAGGAGGAAGCGTTATGCCGGTTATTGAACGGGATGTAGTATTGCAGGGCGT
GGATGAAAATGGCAATCCCACCATCGATCTGCCGGTCACCAGACTGGGGAATATTGAGGATACGGCGGAGGTC
AAGGAAGCGCCGGGGGAGGGAGACTTTCTTCCTGTTATCGATGGCGCTGACGATGGACAGATGAAAAAGACGC
CCTGGGTCAGTATGCAGGGACCTCCGGGACCACAGGGGGAACGGGGGCCCGCCGGTGCGGATGGGGGGCCAGG
GCCGAAGGGAGCCCCCTTTACCTATGAGGACTTCACGGAGGAACAGCTGGCGGCGCTGCGGGGGCCTCAGGGG
CCGGCCGGGGAGAAGGGAGCGCAGGGCCCGGCAGGGGCGGCGGGAGAGTCCGGGAAGTCTGCCTACCAGTATG
CTCAGGAGGGCGGCTATACCGGGACGGAGGAAGAGTTCGGGGCATTGCTGGGGCTGACGGGGACCATCGCCGC
AAAGCTGGATGAGATCAACGGGGAGGTTGTGTGATGGGAACGATCATTCAGAAGCTGGATAAGACGCTGGAAA
CCAAGGCGGCCATCCGGCAGGCCATTGTTGGAAAGGGGCAGGCCGTGGAAGATACTGATCCCTTCGAGTCCTA
TCCGGAGAAGATCAGCGCTATCCAGACCGGCGTGCAGCTGCCGGAGCTGACGAACCCCGGCTCCGCCGCAGAT

TTGCTGGCGGGGAAGCAGCTGGTAGACCAGTATGGGAACCCGCTGACGGGGACCATGCCCCAGAAGTCCGCCG
CCGATCTGACCGCCAGCGGGTCCAGTATCATCGTGCCCGCCGGGTACTACCCCAGCCAAGTCTCCAAGAGCGT
AGTTACGGCAACGCACCCCAAGCCTGCCATCAGCCGCAGCGGCGGTACCATCACCGCCAGTCACGTACAAAGC
GCCGGGTATACCGAGGGCGGGACTACTACGGGGACGCTGTCCATCCCTACCCAGGCAGGTACGACCATTACCC
CGGGAACAAGCCAGAAGACCGCCGTGTCCAGCGGACGCATCACTACCGGTACGGTGTATGTAAAGGGGGACGC
TAATCTCAGGGCGGAGAATATCAAATCTGGGGTTAACATTTTTGGGGTTGCTGGTTCTGTAGAAGCCAAAGAA
ATAGTGCATTTGACGATTGATACAACTTCGATTCATAATAGTAACATAACCATAGTTTATCCTGGATCTTGCA
CTAGTACATCAATTGGATTTCGAGTGATTTCACCGGGCATAATCGAAACATTTGATTTTGAAAAAAACAATGT
ATTTTTTGTTAACTATCGTGACAAATATTCATATAATTTTTCGTTTTCTGGATCTATTATGCACGACGACGAA
CCATATCCGGGACGCATAGATATACATGGATATACAGAATATTTTTATTTTCTGTACATATATGGCAATGCTC
AAATAACTATTAAAAGTTGAGTCGTCCCTGGGTGGAAAGCGGCGGAGCATAACCGGATTTGACAAAACACGGC
GCATCATAGTATAATAAATTCACCTGCCATCCAGGTAGGACGGACGGTGCGCTGTTACATATGGCGGTCAGCC
CATTTCCTCGCGATTTGATTCGGGGGGAGGTGGTGCGGATGTGGAAAAGGAAGCTGTTGAAAGCTCTGCTTAC
CGTGATTTTCACGGTGATGACAGCGATTTTACTTACCACAAAAGCGTGCTGACTGCCCGGCTGGTACCCAGGC
AGTCAGCAAATCTTTGTTGACATGTAGGGGCTGACCGTCATGTAACAGCGCCCTTCTGTTGTTTATTATACCC
GCCCGCCCCGGTTTTGTCAATGACGAACCGGGGCGGGTTTTATGTTTTGAAAAGGAGTTGATCGATTGGAAGC
AATCATCGTGGCGCGCATCGGACTGGCGGGGTCTGCCGTGGCGTATCAGATTTACTTGTATAAGCTGGCATAA
ACAGAAAAGGCCGCCCCCAGGAAGGGGGCGGCGGGGCGCTATTGTGCTGAACCCTGATACATTTCCGTCAGAG
CGTTGGTCAGCACCTGGGAAAAGTTGATCTTTTCCCGGTACACCGTCCGCATACCGGCAGCACCTCCCCCGTG
GTCGTTAAGTTACTATCCCATACAAGCCCGAAATGCGCCCTCGCGCATTTTCCCTCTTGTATATGTCCCCGGA
GTGTGGTACAATATGTTGTACTCAATTTACACACTCTCCGGGGTGTCCGCCCTGCGCAAGCTGTAGGAGGCTT
GCGCAGGGCATTTTCTTTTATCCGAATCTTCTTTTAATGCGTCCCAGCAAATCGGGATCGTCCGTGATAGGTC
CGTATACCCCCGTTCCAAACATTCCGCCCACCGACGGTACAAGCGGCTTGCCCATCATCGGCGTTGAACGTGC
CAGTCAGTTTCACGCCGTCCTTTTCCCCGCCCCCTTCCCCGCGGCCCGGCTCACGCGCCGGCCCGCTCCGCCG
CCGCCTGTTCCGGCAGCAGCGGGCCACACACCACAGAAGCGGCGCCCTCGGCCCACCCCAGCAGGTAGTCCTT
TGTCGGTGCGGACGCTCCCTTGACGACCTTTGCAATCGTTTCCATGATCTTCTTTTCCTTCTCGGACATGGTT
GTCCCTCCTTCTTTCTTTTTCCGGCCTCCTCTGCTATACTGTTTCCGGACGGCGCGTCACCGCCGCCGAAACT
GATAGAAAGGAGCAAAATGTTGTGTGCCCTGATCTACTGATTGCTGTTGATGTGTCTGGCCTGGAAACCCGCG
AACAAAAAGCCGATGTCGAAAAGGCTTTCGAGACTTGCGGCTGGATTTACGACGGAGCCAATGCGCCCAGGTC
TGACCGTTTGACTGGGGTCGCATCCATAGAAACCTACTATTTCATGTGGGAAAGATTTGGCGATCCGGTCTAT
CCACAGCTCCCGCCAGCCTGTCGGATTACAGAAAACCCGCTAGTGTAGGTTGAATACATCCACAGCAATCAAA
GGAAATTCGGGGTCGTAATCCACTACGATCCTGAATTTTTCTTTTCCATCCAACCGCCGCAGAGTAATTCTTT
TCAGCATTTCTACATGGCGCTCGTCGTTTGCCAGGGAATTTTCTGAAATTGGCTTGCTGTCCGCTTGCAGTTT
GTCGAAATGCTCCGCAAACACATCTATAGCCTCCAGCGTGTCCATCCCGTTTCCCTCCTTTCATTTGCCGCTT
TGCCCTCCCAACTCTCCCGCTCGTCCCTTGCATTTTCCACCGTCCTCCCGTATAATCCAGGTACAAGCCCCCG
CCAGGGCCGAGTACATAGAAAGGAGGAAGTCTTTGTGGATGCTCTGAAAAAATTCGAGGACAAGGTTACGAGT
GAGTATGTAACCCATCATTCCTTGCAGTTGATGAAAGACACTATTTCGCTCGCCAACGATTTTGCAGATGTTG
AAGACCCCCGCAAAAGTGTACGAACACCAGATTGCTATTTCAGCTCAACACCGCTTTGTTTCACTTGCGCTGC
GAGAATATCACTGTATTCTGTCGCAGTACCTATCGAAACATGGAATAACCTTGCCAGACCTAGAATCACTGAT
TTCCGAAACCTATCAGGATTGATGTCTCCCTGCCGCTCCTGCACCTCCGGCGCTGGGGCGGCACTCTTTTTTT
CGTCCACCCCGTTTCCCTCCTTTCGTTTGTCGGATTACGACAATAATAAATCATTATCCGACATTTGTCAATA
CCCAAAATGTCGGTTTCCAACTTTATTGTTGACATCCGACATTTTGCGTGTTATGATTGGCAATACAGAAGGG
GGTGAACAATTTGACAATAGGCGAGCGCCTAAAAAATCTACGCAAGGCCCTGGGCCTCACCCAGCAAGAATTT
TCGGACAAACTGAAAGTAAGCCGGAGCAACATTGCCACCTACGAAGTCGGCAAAAACAATCCCGCCGAGGCGG
TTATCAATCTGATCTGCCGGGAGTTCAATGTATCGGAAACATGGCTGCGGACCGGCGAAGGGGAAATGTTTGT
GAAGCGGACCAGGGACGATGAACTGGCCGCGTTCATGGATGAACTACTGGCGGAGGAAAGCGCGGACTTCCGG
CGTCGGCTTGTAACCGCGCTGTCCCGGTTGCGGCCGGAACAATGGGAGGCGCTGGAGGCCGTCGCCCTGGAGC
TGATGAAAGACCCCGCCGCCCCGGCCTTTGCCCCCGCCCATTTCTCTACCATTGAGGAGGAGGCCCACGCCGA
GGCGGAGCAGCAGGCCCAGCTTATTTACCAGCAGATTCTTTCTGAAAAGAAAGCCG
>MP_1_2_20_S60_clean_1_73531 flag=1 multi=4.0000 len=9045
CAGCGATGGGGACCGCTTTTGCCGCCTCTTTTGCCAAAAGCACACAGGAAATCAATCGTTTTTACAACCTCTC
GAACCAGACAGGCGCCTCTGTCCGATCAATCAACAATATTTCATCTGCGTTAAAAAAGGTCGGCGGCAGTGCT
GAAGCGGCTCAGTCTGCGATGCAGAGCTTATCTGACAAACTAATAAGATTCCCGGCGCTTGAAGATCAGCTAA
AACGTATGACGGGAATGACCGACATCAAAGATTCGAACGGCTACGTTAAATACGAAGAGATTCTTTTGAGACT
CCGCGATCGGTGGAAGGATATGCCCGAGGCGGTTGCACAAAACGAGGCTGCACTACTCGGACTTGAAGGGATT
TACTCTTCCCTCATGAAACAAGATTTTCCTGCTGAACTTGAAAAAACGAGCAAACAACAAGGCGAGCTTACCG
ACATGATTGATAAATCGGCCGATTCCGCTCACCGTCTTGCGAATGAATTCTCTAACACATGGAAATTATCTC
AAACGGGGCAAAAGCCGCCGCTGGACAGCTCATTGAGGCGATAGGACTCGATAAAGAACTAGCGAAAGTTAAC

GACAAACTATCTAAAGGTTTGCCTGAAGTGCTCAAGACAGAGAAGTACATCTGGGACAAATCGAAAAATCCTG
TTGATTGGGCCTACAACATGTTATTCAAAGCCGATGACTTACACGAAGATAGGCTCTACGAAGAGCACAAGTT
AAGCGATGAGGAAGTACAAAAACGCCTCAGGAAAAAGTACACAAAAATCTTACCGGTTCTAAGCGATGAAGCC
GAAGAAGGCGTAGACAACATCGAATATTTCGATCAGAAAGGCTACGAAGATGAACTTGCTAAGTACCGCAGTT
CACAAAAGGATAAGCAGATGACGACACTACCGGCAACTCAGTCAACGCAACAAGAAGTGCCTGAAAAGATGAC
CCGTGGACTGCGCAATAACAATCCTGGAAATTTACGGCCCACTTCTATGAACAGCCCGCAAAATGGTGGCTTT
CAAGTGTTTCCTTCACGTGAGGCAGGCTGGGGCGCATTAGGACGACAGTTAAAAGGTTATGCGAATGCTGGCC
TAGAAAACATCTACTCCATCATTTCAAAGTATGCCCCTTCAACAGAAAACGACACAGCTGCATATATCAATGC
TGTAGCCGGCTCCATGAGCCAGCGTCTCGGCACAGTTGTAACGCCGGATGCCAGATTGGATTTGAAGGATGCA
AGAGTTTTGAAAGCATTAATGATGGCTATTACAGACCACGAAAATTTCAAAGGCGCTTCCCGCTATTTCGAAG
GTCCTTCTTTTGATCGTGAAGTGGGTGCCGCCTCTCAGTCTGAATGGAAGAGCAAAGTGGTAAGCGACAGAGA
CAAGTTTGCTTCTAATGGACAGGTCACAGTCAACCAGAACATCACAATTAATGGAGCAGACAATCCCACGGCT
GTAGGTAAGGCTGTAGCCAAGGAAACTCTTCTTGCTCAAAACCGATACGGCACCAGAAACATTAGTTAGGAGG
ATCTATGCCTTCTTTGCCATACAGCCTCGAGGCATTACTTCTCGGAAGAAAACGAGAATTTGCGGGCATTATT
CCTGATGTAATCATCAGTGAAGAGCATGAAAATGAGGTAATAGTTACCCGTCATCCGGTGGATACAGGGGCCA
ATGTCGCAGACCATGCTTACTGTTTGCCAGCGGTCATCAACTGTCAATTCGGATGGTCAGACTCTTCCAGATT
GTTAAATTCAATCTTAGATTTTTCAATCTTCAAAGGTCTGACCACTACAAAAGATGTTTACGAAAAACTTCTT
GAATTGCAGGCCAAACGAGAACCTTTCGCGCTATCTACGGGGAAAAAACAATACCCGGCGGTAATCATCACCA
AATTAAAAACGACTTCAACAGTCGACACAGAAAGCGCATTGGTGGTTGACATTACCTTTGAGGAAATCCGATT
TGCAAAAACCAAAGAGGTAACTCTTCAAGAGGCGCAACAGAAAAATCCACAGCAAACCGCATCGGTCAATCAG
GGCGGAAACAAAACTTCCAGTTTGACCTCGGCAGGCAACCGTCCAACCGCAAAGTGAATATATGACATACCAA
ATTCCATTAGGTACAGGCTCTCAGAGTTTCTCAACAAAATTAGGAGAGCAGACCTACAAAATAACGATTATTT
ATCGGCAGGCCGATGGCGGCGGTTGGTTTCTCGATATGGAGACGGATACAGAGGCAATCTGCGGGATTCCGCT
TGTGACCAATGTCAACCTGTTGGAGCAATTCAAATACAAGTCTATGGGTGCAGCACTGTGGTGCGAACTATCC
AGTCACAGCCGAAAAGTCTACGAGCCAACCTACTCCGACATGGGTCGCACCCTTAACCTTTATTGGAGCGATG
AATGAGTGAAAATAAAAACAGGCAGTGGCTGCGCTATTTTCGCCTCGTAATCGCTACTGACAAAAACAATCAG
CAGGCCATCGATTTAAGTGAGTTCCGGTGCAAATTTCATATTTCTCAAGCTGTCATCGGCAAACCTTGCACGG
CAGAAATCACGGTTTACAACGTCTCTAAAGAAACTATCGACAAACTTGGTATCGGAACCAACAAAATCGAAGA
CAGCGGCATGAAGGTGATCATTGCGGCTGGATACAAAGACAATCACGGCATTATTTTTCAGGGCGACCTTTGG
TGGAAATCAACCGGTCGAGAAAGCGAAACGGAGACATTCATGAAGCTAGTCGCTGCTACTGGAGACAAAGCAC
GGCAGTACGCAGTTGTCAATGTTTCAGTTGCTAAAGGTGCAACCCAGCAAACCATATTCAAAAAAGTCTGCGA
GGCTATGAAGGCCAAAGGTGTTGACGTTAAAAATGCCTCAGTCCCGTTCATGGACACACGTCTACCAAGAGGA
AAAGTTCTTTTCAAAATGGCCACAGACGCCATGAATGGCATAGCTGATACAAATAATTTCGATTGGGGTTACG
GAGTCGATGGATTAGTAGCTATTCCCAAAGAACCGGTATTCAATCAGAAAGAGACTGTAATCGTACTAAACGC
AGATACAGGCCTTATAGGAAGACCGGAATTAGATGAGGAAGGTTTGGATGTGCAGGCTCTTTTAAATCCTAAA
TTGGAGATTGGAACCCTCATTCAAATTGACAATGCATCAGTGCAGAGGAACTCATACGACACCACTGTTTCTG
AAGGCGCCTATCAAAAAAACATGGCTGTAACCGATGATTTTCTTTCGGCCGATGGAATCTATCGAGTGATTTC
ACGTGAGCACATCGGAGACACCAGAGGCGATGAGTGGTACACGAGTCTGATTGTCGTGGGCGTTTCTTCGGCT
TCTCAGCCTATCGCACCTTCTATATTCACATTTACATCGAACTGAAAAAATGGATTCAACTTCAAATATTTTT
GATCCGAATAAGTTCACGGATAAAGCGATTAATAGTCGCATGACTATGGTGTGGACCGCCCTCCCCGGAATTA
TTCAGAGTTTCGACCCGCAAGCATTGACCTGCGAGGTACAGCCGGCTATCAAGGGGAGAATGACAACCGAAAA
AGGCATTGAGCTTGTTAACATGCCGTTGCTTCTTGATTGTCCCGTGGTGTTCCCTCATGCAGGAGGTTGCTCT
CTAACATTTCCAATTCAGCCGGGCGATGAATGCTTAGTTGTTTTTTCTAGCCGTGCGATTGACTTCTGGTGGC
AGTTAGGAGGTATTCAACCTCCGGCTGAGGCAAGAATGCACGATTTGAGTGACGGATTTGTAATTCCTGGACC
ATACTCTCAGGCCCGAAAAATCTCAGGAGTAAGCACATCGGCTTTGCAGATCAGAAGTGATGACGGGACCGCA
TTCATTGAAATCAACCCATCGTCTCACAATGTCAAATGCCAGACTAACGGAGATTTTTCCGTCCAATGCAAAA
ACTTTGCGGTTGAGGCGAGCGCTTCGGCAACGATTACTTCGCCTGCAATAGCTCTTAACGGTGCTCTGACAAA
CAATGCTTCTACTGAGGCTCAGATGACTGGCGGATTGAATACAACGAAAGACGTGACCGCTTCAGGAATTAGC
CTCAAATCTCACACCCATTCAAATGTTCAGAATGGCCCGGGCAATACAGGAGGCCCGCAATGAAAGTCAGGAA
ATTAAACGCAGATGGCGACATGTGTATGGGTCACGGCTCTGCCGATTACATCGAAAACACGCCGGAAGCTGTA
GCACAGAATGTTATGACCAGATTGTCTCTCTGGAGCGGTCAATGGTTTATTGATACCGATGAGGGCACTCCTT
ATTTGCAGGAAATTCTAGGGAAACATGATGCTGTTGATTTGCTTATCAAACAGCGGATTTTAGAGACACCCGG
GGTCACACAGATTGAACAGTTTGAGGCTATTTTCGACCCTGACACCAGACGGCTGTCAATCACGGCGGAAATA
GATACAGAGTACGGAAAAACGACCGTAAATGGAGACATTCAATGATAGAAGATCCTGTATTCAAAGTTACTGA
GGCAGGCATTTCAGCTCCTTCGTACGCAGAAATTTACGAGTATTACAAGCAGCGGATGCGTGAGATTTTCGGA
GACGATATAAACCTTGATGCTGACACTCAGGACGGTCAGATGGTAGCAATGTATGCTCTTGCATTATCGGACG
TGAACTCACAAGCTATCGCTGTATACAACGCCTACAATCCGAGTACCGCAAAAGGCATAGCCCTCGATACAGC
TGTAAAAGTCAACGGCATCCAGAGGCGCTCTGCATCCCACTCCCAGGCCGATCTAAAACTCATCGGTCAGGCA

GGCACTCATATCATTTCAGGAGTTGCGTTAGATGAACAGGAAAACAAATGGCTGCTGCCTGAGGATGTAGTGA
TTCCGCCCAGCGGAGAGATTGTTGTAACTGCCATTGCGGAGGATGAGGGAAACATTAAGGCTGCACCCGGTGC
GATCAACAGAATCGGGACTCCGACACTTGGGTGGCAAACAGTCACTAATGCCTTAGCGGCCGAAGAAGGCGTG
CCTGTTCAAACAGATGCCGAACTGCGCATCCAGCAGACAAAAAGCACCGCTCTCCCAAGCGTTTCGCTGTGGG
AGGGGATTGTCGCCAGTTTATTAACAACAGATGGAGTAAGGAGAGTCAGCGGTGTCAAAAACGATGGAGATTT
ACCGACAGATGAAGGAATTCCCGGCCACTCCATTGCGATGATTGTGGACGGAGGCGATGTAGCCGAAATTGCA
AAAACCATATTCTTAAAAAAAGGCGAAGGCGTTGGCACCTATGGTTCTACCACTTACACCTATCTGGATACAT
ACGGATTTCCAAACAAAATTTCATTCTCACGTCCGACCAATGTAGATATTTTCTGCGAAATCAAAATATCCCC
CGCTCGAAATTTTCTCTCTACAGACGAGGAAGAAATCAAAGCCAGGATAGTGACTTACATCAATTCGTTAGAA
ATTGGCGAGAGCGTGAATATTTCCCGAGTCTTAGCCAACGCTGTAAAAACCGACAACGGTGTCGTTGACACGA
GGTTTGTAGTCAATGACATTTCTCTAGGTGAATCGTCTACCCAACTTACAACTGCAAGCGTAGATATTGAGTG
GAACGAGGCAGCTATTTGCGAATTGCAAAATGTGACTGTAACAGTTGAGGATGTATCATGAATCATGAAAATC
AATATACCGAACTGATTGCAGGGGCGCATTTCGAAAAACCAAAATACTCAAGGTTTATTTATGAGATAACGGA
ACCATTGAATATTGCTAGGAAAAGGCTAGCGCTTCTTTACGAGAATTTCGATGTAGATACCGCAGTCGGAGTC
CAGCTAGATGCTATAGGGGTCCGAGTTGGAATATCAAGACATTTGCCGATGAAACTCATCGGCATTTATTTTG
CCCTCGATGATGTCGATGGTGTGGGTTTTGATAGGGGTGTGTGGAAAGGTAAGTATGACCCTGACGATGGCAT
TGTCTCTTTGGACGATGAAACATACAGACTCGTAATCAAGACCAAAATCCTCGCCAATAAATTCGATGGCAAA
AACCAGTCAGTTCCTGAATTTATTAACACAGTGCTCGGTTACTTTGGCGTACCGGCAAAAATTTTCGATTTTC
AGGACGAGCAAAACATGCACGTAGTCCTGAACATGACTAAATCCGAAACACCTCCCATTGTCTGGGAACTTAT
CAGCCGCAGACTCATCGACATTGTGGCGGCAGGTGTCGGAATGCAAATAGTCGATAACGAACCGTATTTTGGA
TTCGATTATGAAACAGCCTCTGTCAAGGGTTTTGACGAGGGTCATTTCTTCCCGTTTGAAAACTAGTTAGCAG
GTAAAAGAATATGTCAATAATGAACGAATTCCTCCCGTTTGCAGAGGCCGAAAACGCAAACTTGATACCATTT
TCAGAATGGCAAAACGCAGCCGCTCGATTGAGCGGCTTTTCTTCGGGTATTGCGCGCTCCAACCAGATCAACC
GCGCAATTGCTCAGGGTGCAAACATAAGCTATGCCATCGCTAAATTCATTGAAAGAACTCTCAAAGAAGACGT
CTACGTTAGCAACCCGGATCGTCTCGTAGATCAGCTCTATAGAGCGATGCTTGAGATGAGTTATAGAGCGACT
CCTATCGGTTGCATTCTCACATTCCCCGTTCAGGTTGAAATTGAAGGCTTTGTCGTTTCAAACTTTGGTGGCG
ATTTGTCACGTATTGATTACGACAAATTATTTGCGGTATACGGCACAAAATTTAATGTTTCCAGCACACCAGC
AAACAAGTTCGGTATTCCAAACCTTGCATACAACTTCCTTGAGGTTGCAGGCCAGCTCGCAGAGATCGGTTGC
TTCGTAAAAGCTGGCTTACCGAATATCCCGGGGAGCCTGGAGGTTTGGACTGGAGTCAGAGGTTATTTTTCAA
TTTCATCTAGCGGTGCCTTTTATTCAAGGAACAATGGTGAAGCACCGATCGTCTTAAATGGCGGCATCACTTA
CGAAGGTAAGACAGATAACCATATAGATTTTTCTCCGGACGTTTACAGCTCGATTTACAAAGTCGGAGTCAAC
ACTGTTCAGCCGCCGGCACTAAGATCATTGGCGTTAATACGCGCGTATTAAACAGAGCGAACGAAGAGCAGAC
ACTTGTACTGTGGTAGCACTTCCGTAAATCGGATTTAAATCTCCGGCATTAAAGGAGAAGGTAGTATTTTGCC
AAGATGCGCCCGTTGAATTTTTAGTGGTAACAATGTAATCAGGAAATCTGGCGATCATTGAATAAGGCGACTC
TGAGTCTTTTTGGTTGTATGCCAAAACACTTAACCAGCCTCCCGGGATATTCGGAAATTGACCTTTGTGCGCC
TTTTGCTAGAATACGTTCACCTACTCTGAAGGACATGGCAGAGAAAGGGATTGGAAAGTTGCTGGTTATCTTT
CCATTCTGTGAAGAAACCAAGCAAAACGGCTCAGAACTACCAATTCCGAGCCGCCTTATAAGGATGAGATCCA
TGTTCCAATTAAAATCTCATGCCTCTAGTATAAAGCGCATTGTCAGAAAAATCATTAAATTGATACTGACAAT
GGTGCTCATGTTGTACTGCTAGGCACTAAGGTAAAGAGCCCCAAAGCGAAAGCTGAGGGGTTCAAGTGTTTTT
AGACCGAATATCTCGGGTGCGGGAGTCGAATATATGGAACTGAGCAATTTCGCCACTCCTGAAGGAGCTTTCA
GAAAAGCGAACTGGAATTCTTATTACGACATCGAGTCCTCAATCCGCGAACAAGAAGGCGGGTTTAGAGAAAC
CTCACTAACATTTTCAGCCGGATGGTCAAACGATATCTATAAAGATTCCATTTCGACTGTTCAACCATCGGCT
ATCCAAGCACTTGCCTTAATACGCTCTTATTAAACATAAAGAGCGCAAGGCCGATGGTTGGTTTGTGGTCGAG
CTACCATAAGTAGCGTTATACGTACTCGGGTCTAATTTAAAACTAACCGAGGCCGTCTCCACTTGACTTGTAT
TACCCGCAAAAACGTATTTATTGCTTCCTGCTGCACCGTCTAAAGCAGTTACAGCTGATCCGCTGGAGGCAGT
GGCGTAGTTGTCGTGTGGAATACGCCCAAAATAAATTTGAGCGGGAATATTCGGGAATTTTCTTTGAACTGTC
AGAAGGCCCGATACAGCTTAGTGGAAGTCGATTTTTACGCTGTGCCGGGCACATTATAAAAAGTTCAGCTTTT
GGAGCTTAACTTATTTATTTCTCAGGTTTTTCTTTGAAAATCTGAGCACATTGAGCACACTGCATTATGTAAG
CGTGCCAACCAAGCATAACTTTAGACCTAGACGCAAAATAATCGCCTCTTTGATAAGCACGTACGACCTGCGA
ACCAACTAAATGAGCTAAACACGCCTCTGCAACTTCAAAAGGTACATCATTGTCTGCGAACCAAGACCTACCA
ATTGAGCGCAAACCATGAGCGACAAGACGGTCTTTGAATACCTCTTGTCCGTGGAGCCATTTAGCAAGAGCTT
GAGAGGAAATTGATTTATTCGCATTGATGGCCGGAAAGAGGAAAGGCGAACGCTTATTTTTGCGTATTCTTTT
AGCCTCGTTTATTAGGCGAATTAAGTATGGCGTGATCGGTATCCGATGCACTCTTTTCATTTTCATAAATTCT
GCAGGAATGGTGATAGCCTCATCGCTTATCCATTCCAACCGGATAGAAACAACCTCTCCCGGCCGAAGCAATG
TCGCCAAAGAGAAATAAAAAAGAACACGGTATTTTGCCGGTGCTAAGGATTCAATTTGCGAGACAACGATAGG
CAGCTCTTTCCAGTCCACTGCCGGCATATGTTTCACGGACGGGACTGCGAATACTTTAGTGATCTTTGTTAGA
GGATTTGAGTGAAGATATCCAGCATTAACAGCCATGTCGAATATCTCTCTTACTCGCATTAACAACCTTTTTA
CTGTTGACTGTTTGCCTTCTTTTTCGACTGGTTCGAGAAGTTTAATTAGAACCGGCGGAGTTATCG

>MP_1_3_20_S79_clean_1_166317 flag=1 multi=5.0000 len=6337
ATTGAGGCTATGGACCTTGGAGACGAGGCCGCAGCAGCTGGCCGGGCCACCATCCAGGGGTACGTTGATGCGG
CAAACGATATGCTCCCGGCGGTGCGCAATGCGTATGCGAATTTGGCACAGGCGGCGCAATCTGCAATGGGATA
TAAAATGGTCTATGTCCCAAGCTCCAATGCTTACGTTCCTCAGCGCGGCTATGCCAGCGGCACAGAGAACGCA
GAGCCAGGCTGGGCCATGGTGGGCGAGGACGGCCCCGAGCTGATGTTCTTCAACGGCGGGGAAAAGGTTCTGG
ATGCCTCCCGGACTTCCGCCCTGCAAGCGAGGCCGGAGCCCGCCCTGTCCGCCATGGTAGCGGCACCCGGGGG
TGGCTCCTCCTCTGTCCAGGTCGTGTTCCAAATCGCCGGCAACGCCACGCCGGACACGGTTCAGGCCTTGCAG
GACTACGGGGACGACTTCGCCGAGCGGGTGATGGAGGTGGTGGAGAACGCCGCGGCGGATGCCGCAAGGGGGG
CATACCGATGAGAACCTACACCACAGTACAAGGGGATATGTGGGACAGCATCGCCTATAAGCAACTGGGGGAC
ACGGCCCATACCGATAAGCTGATGAACGCCAATCTGCGGTATCTGCGATACTACACATTCCCCGCCGGCATCG
TCCTGACCTTGCCGGAAATCGGCGAGAGCGTCAGCGACACACTGCCTCCCTGGAAGCAGGTGGGACAGTGAGC
AATAAGAATTTAGCCCGCCGGGCGGCTGTCGGGATCTCTTTTGCCGGCACCGACATCACCAAATCCATCAAGC
CCTACCTCAAAGCCTTTACCTACACCGATAACGAGGCGGACGAGGCGGACGACATTCAAATCCAGCTCCACGA
CAGGGACACTATCTGGATGGAAAAATGGCTGAACGAGGCCATTGACGCCGCCTCCGCCGCAAAGCTGAAGATA
GATTCGGTGATCGTTCGGGAGAACTGGCGGGGGGACGGAAAGGACGTGGTTCTCCCCTGTGGAGAATGCGAGT
TGGACACCGTAGACTACTCCCTCCCCCCGGCGGTCATTACCATCAAGGGAACGTCCCTGCCCTTCAGCGCACA
GATCAGGCAGACCAAGAAGAACAAGGCCTGGGAGAACACCACGCTCTCGGCAATCGCCAACGAACTCGCCGGG
GCAAACGGCATGACTTGTATGTATGAGTCTGCCAACGATCCGTTCTATAAGCGGGTGGAGCAGGTCGATGCCA
GCGACATTGATTTCCTCTCCCGCCTGTGCCACGACGCGGGCATTTCCCTGAAAGCCACCAACAGCATCATCGT
CCTGTTTGACCAGGCCACATACGAGGCCAAGGGTGCGGTTATAACCGTCAGGAGGGGCGATGGAAGTTATACC
AAGTGTAAACTTACGGTCGGTGCGCCGGACGCCCAGTATTCGTCCTGCCGGGTCAGCTATGTAGACCCGTCCA
CAGGCAAGTGCATTGAGGCCACCGCCAAAATCGAGGACTACAACGCCGACGCCAAAAACAATCAGCAGTTGGA
GGTAAAGGCAAGGGTGTCCTCGAAAGACGAGGCCAAGGCCCTGGCGGAAAAGCACCTGCGTCTGCACAACAAG
TATACCAAGTCCGCCACCTTTACCATGCCCGGCAACCCCGCCCTGGTGGCTGGCGTCACCGTCATGCTGGAGG
GCTGGGGCGGCTGGGACGGCAAGTACATCATCACCCAGGCCAAGCATACGGTCAGCGATTCCGGCTACACGAC
CCAAATCAAGCTGAGGCGGGTATTGGAGGGATATTGATGGACGTTGAAAAAATCCTGGCGCAGTTGGTGCGCA
TCGGCACCGTCACATCCGCCGATCCCGTCAAGCGGGTGGCCCGGGTGAAGTTTCAGGACACCGGCATGACCTC
TGGCCTGCTCCACGTCCTGGCCTCCCGCCCATACATCCCGGACTACGAGACAAAGCCCCAGCGCACGGAGTTC
GAGGCAGGAGGCAGCGGCGACGCCGCCTACGAGCGGCACAAGCACGATTTGGAAATCAAGCCCTGGATGCCCA
AGGTAAACGCTACCGTCCTGACGCTGTATCTCCCGATACTCGACGCAGACGGTTTTATCCTGGGGGAGATCGG
GCCGCTGGAAAAGCTCAAACAGATGGAGTAGGGGGTATCTTAAGGCAATTATCGGCTGTCTCGGGGACGTTGT
GTTCACGGTATCCGAGGCCACCGTGAGGACGCTGGACAACATGACGTGGTCCGGCTCCGCCCGGTATGCCGTC
CACAACCGGCACCTGACCCACGCCCTCACGGAGTTTGTTGGGCTTGACCCGGACAAGATCACGTTTGATATTA
CGCTCTCCACCGACCTTGGCGTTGACCCTATCGAGGAGGTGGTGAAAATCTGGAACATCGAGCGCAGCGGGAG
GGCGGTTCCGCTGACCGTGGGCACCAAGGGCTACGGCAAATACCGATGGAACATCACCAAGCACGAAATGAAG
ATGAAAGCCCATTTCCGCAACGGCGACATTCACACCGCCACGGTGTCTGTCAGCCTGCAAGAGTACCTGGGAG
GTTGACGCTATGAGCTATACAGTCTCCGCAACAGACCTGGGACGCCTACGGTTCAATGAGCTGGAAACCGTGA
ATTCTGTGCTTCAGAACATCGCGGTCATTCTCTCCACGCCAAAGGGGACGGTTCCCCTATACCGGGAGTTCGG
CCTTGACTGGAAGTACCTCGACAAGCCAATGCCGGTGGCAAAGGTGCTGATGATCTCCGAAGTGCGGGAGGCC
GTGGAGCGTTGGGAGCCCCGGGCCACCGTTCTGGATGTGTCCTTTACGGTAGACCCGGCACAGCCCGGTACTC
TGATACCGACAGTGGAGGTGGAAATAAGCCTTGAGTAGAAACGCAGAATATCAATTTGTCCCCACGGACACAT
CGACCATTGAGGCCCTGCTGGTGGCGCTGTGCGAAAAACTGACCGGGGCAACGATCCAGCCCGGGAGCCCTGA
CCGGCTGCTGATCCAGTGGGTCAGCAACATCATCGTCCAGGAGCGGGTGATGAACAACTACACCGGCAACCAG
AACATCCCTAGCCGTGCGGAGGGGGAGAACCTGGACGCCCTGGGCGAACTGTTCCTGGAGCATATCCGCCCGG
CGGCAAAGGCGGCAACCTGTCAAATGCGGTTCTCCATCTCCGAGGCGCAGGAGTCCGCCGTCCTGATCCCCGC
CGGTACCCGCATCACCGACGCAAGCGGTACGCTCACCTGGGAAACGGTCAAGGACGTCTATGTCCCCATCGGG
GAAACCAGCGTGGAGACCCAGGCCCGGTGCCAGATTGCCGGCACCGCCGGCAACGGCTATTCCGCCGGCCAAA
TCAACGCCCTGGTGGATCTGTACGACTACTACTCGGAGTGCACCAACATCACAGAATCGGACGGCGGCGCCAA
CGAGGCGACGGACGAGGAGTTTTACGAGCTTATGAGAGCATATATGGACGGCTACAGCTGCGCCGGGGCCCGG
GGGAGCTATGAGTATTTCGCCAAGCAGGTCAGCACGGAAATCGCCGATGTTGTGGCGAACTCCCCGACCCCTG
GTGTTGTCAAGCTCTATGTCCTCATGGACGACGGGACAATCGCCACCGAGGAGATGAAAAAGGCGGTGCTGGA
CGCTTGCAGTGAAGATACCAAGCGCCCCCTGACCGACCAGGTATTCGTGGAGGATGCCGAGGTCGTCCCCTAT
GACATCAGCTTTACCTACTACCTCCAGACCGGGCGCACCAAGAGCGCGGCGGAGGTCGCTGCGGCGGTGAACG
AGGCCGTGGAGCAGTATAAGGCGTGGCAGCACGCAAAGCTGGGCCGGGACATTAACCCGGACGAGCGGAGGGA
ATATCTCTACCACACCGGCGTCAAGCGCATCGACCTTGTATCCCCGGCCTTTACCGTCCTCCGGGACGGCAAA
AACAAGACCGTCCCCCAGGTGGCACGGCTGGGAACGGTGACGATCACAAATGGGGGGTATGAAGATGAATAAC
AGGGAGCCCGGCGCCGGCCACGGGCTGACCAAGGAAAACCTCGTGGCAACGCTCCCTGTCGCCCTGCAGAAAG
ACCCGTCCGTGGTGGCGCTGGCGGAGGCCTTTGCCGAGCTGCTGGCCCGGCGCCCGGAAGAAATTGACCGGCT

GAGGATCTATCCGGCCATCGACCGGCTGGACGAACGCCTGTTGGACATACTCGCCCACGACTTCAAAGTGGAC
TGGTGGGACGCCGACTATTCGTTGGAGGAAAAGCGCCGGACGCTGAAAGACAGCTGGCGTGTCCACAAGATAC
TGGGCACCAAGGCGGCGGTCGAGCGGGCTATCTCCGCCATCTATCCCCACACACAGGTGCTGGAATGGTTTGA
GTACGGTGGGGAGCCGTATCACTTCCGCTTGGATATCAACATCACCAACGACCACATCGACTCCGACAAGCAG
CGCCGTGTCCTGGAGCGGCTGAACTACTACAAGTCCCTCCGCTCCCACAACGACGGGGTGACCTACTTTGTGG
AGGCGGAACCGGCCCTTGCAAAAGCGGCCTCCAGCGTTCCCGGTTTCAAAGAAACTGTCCACGTTCCCTTGGA
GCTGCCTGTGCCCATCATCCGGCCCACAGCAAGCGCCCGTGTCGGGGTTATAACCGGCCTGTGGGAGAGTGCG
GCGACACGGCTGGAACTTCCTACCCCCATCATCCGGGGTGTGGCTATGGCCCGCACAGTCGTCTCTACTGGCC
TCTATGAGACGTTTGCCGCAAGCCTGGATTTGACCGTCCCCGCGCCGCGGCGGGTGTCCCTTGCCCGCATCGG
AGCGTCGGCGGGGATGCAGGAGATTTTTGTCGCCCGTGTCGTTCTGCCTGATACAGACCCGCCGAGGGGTACG
GCGCACTTCCGAACCGCCGTGTCCAGCGCATGGCAGGAACGCTACACCACCGGGGCGATCCCCCTTACAGCAC
AGGCACCAACCGCCGCCGCTTCAGCTCCGGCCCGGGGCGCGGTGACGGCGAGGCAAGAAACCGCACAGACCAT
AATCAATCTTTAGCAGGAGGTAGACCATGGAAGAAAACAAGGCAACGCCGCGTTGGTCGAAAACGGCAGTAAC
CGACATTGGCACGGCCCTGCTGGCCGAGTTCGCAGCGGGGCGCATTCTGAACATCACCGCCGCATACGGTTCC
GTCGGCGCAGACGACAACCTGATCGAGTTGGAGGAGCTGCCCGACGGCCGCGCCCACCCGCTGACCATCGAGA
GCGTCACCCGGACGGACAACAGTGTGACGGCCTGTATCCAGGTGACGAGCCTGGGCAATCCGGCCCCCTACAA
GATGGACAGGATCGGCCTCTACGCCATCACCAAAGACCCCGGAGAGACGCAGGAGCCGGACGGCCCCGGTGGC
GGCGTCCTTTTGGATGATAAGCTCCTGATGGTGGTCGAGGACACCGAGGACGAGCAGGGCCGCAAGGGTGTGA
CCATCCCTGCGGAGACCGACCAGCTCTACACGTTCAAGCTCTATGCGGTCTTGACGATCACGAACAAAGACCG
CCTGGAGGTCAGCGTGTCCACCGCCGGCATCGCCACCCTGGGGGCCATCGACGACGCCATGGAGAAGCACAAC
CAAGACCCGGAGGCCCACCCGGGGATGATGGAGAAGTTTGCCGGGGAGCATAATGACGACCCGGAGGCCCACC
CCGGCATGACGGCCCGCATCCGCGCCACGGAAATCGCCCTGAACGGGAGTGAGACGATCCTTTCCAAGAACGG
CGACCCCACCACCGAGACGGAGGGCGTCAAAGGGCAGCATTACATCAACCTGGATACCGGGGCTGAGTTCGTC
TGCAACGACATCACGGACGAGGGCTACATCTGGGGGCCGGTTGACATCAAGACCTCCATGCGGGATCTGCTGG
CGCAGACAGCGGAAACCGCAAAACAGGCAAAGGACGTGGCCGACGGCGCAGCGCAGGCTATCGCCGCCGTCCA
GAACACCATTTCGGTGATCCCCTCCCAGTCCGGCAGTTTGACCTACAACGGGAGCGCCCAAACTCCGAGCTGG
AACAACTACGCTGTCGAGATGATGGAGGTCACCTACGGAGATCCCGACGACCCGGATGCCAGGATCACGGAGG
CCAATTTCCAGGGGCAGACTGACGCGGGCACCTACACGGCCTACTTCAAGCCCAAGGGCGACTACACCTGGGG
CGACAAATCCAAGACGGAGAAAGCGGTTCCGTGGAGTATCCAGCGGGCCACCATCACCACTGCCCCCAGCGTG
ACCGGGACGCTGACCTACAACGGCGGGGCCCAGACCCCCACATGGCAAAGCTTCAACTC
>MP_1_3_23_S3_clean_1_49292 flag=0 multi=63.2845 len=6036
ATGCGCTAAACAATCCCCAGACCACTCAAACATCCACAATCCGAATCAACATGACTGACTTTTTGCATCTCCC
TTTGGTGGAAGTCCTCAAACTTCTCGTAAGGGGGTCGGTGGAGGAATATCTCGCCGAAAAGGAAGCCAACGCT
TCCGCACGCGCCGTTACCACGGCAAACAACGAATGTGAATCACTTGACATGAAGGGGGCTTTGGAGTTTCTCA
ACGCCAACGGCTACCCGATAAAGAAAGGACAGCTCTACAAGGAAACGAGCGCGGGAACCGTGCCGTTCCAGAA
ATTCGGCTCCAAGCTGCATTTCAGAAAATCCGAGCTGTTGCAGTGGGCGGAGGCACGGCTCATCGACGGAAAC
GCTGTCGGCGTACTCGTACCCGCCACAATCGTGAAAGGAGGGAGGCGATGAAAAAAGAAGAAAAAGCCGCAGA
AAAGACCTCCCGCAATCCCTTTCCCCTTGATGTCCTTCCGAGAAAGATGCGTGAAATCATAGACGAGGCGAAC
ACGACCCTCGGATTTCCTAAAGACTATCTCGCCATGTCGATGCTGACGGCGGTTGCCGCCGCTGTCGGAAACA
CACACAACGCCGAACATATGGCAGGGTGGCAGGAATACTGTATTCTTTTCGTCGCACTCGTAGGGCGTCCGGG
AGCCAACAAGAGCCACCCGCTGAGCTTCGCCATGCAGCCGCTCATAGATTTCGACGCGGAGCAGTCTGCAATC
TTCTCCGAGGCTATGAAACGCTATCTCGCGGCGATGGAGCTGCCGCCGAAAGAGAGAGCCGCAAACGGCTATG
AAACAAACCCGACCGAACCGAGGCGCGTCCGCTTCACTATGCAGGATGTAACGCCGGAGGCGGTACACCGCAT
CCTTTCGGAGAATCCGAGGGGGCTGTGCCTCTATGCCGACGAGCTTGCCGGGTGGTTCAAGAATTTCAACCGC
TACAACAACGGCTCCGAATCGGAGTTCTGGATGTCGGTGTTCAACCACAAGGTAGCAATGTCAGACCGCAAGA
GTAGCCAGAGCGGTGTGTTCATCCGCAATCCGTTCCTCTGCGTCATCGGCACGATCCAGCCCAAAGTGCTGGG
CGAGCTTGCCGCAGGGAACCGCAATTCAAACGGTTTCACCGAGCGTTTTCTCTATGTGGTGCCGGACGACCAG
ACAAAGGCGAAATGGAACAGCGAGAGAAAATCGCCCTCATTTGACATCGTTTCGGCGTGGCGTGAGGTTGTCG
GCAAATTGACCGGGACTGTTCCTGCGGCTGACAGCGGAGGCGACATACTGCCCGAAGATGTGCCGTTCTCGCC
GGACGCACTCCAACGCCTCTACTCATGGCAGAACGAATCGACCGAGCGGTGCAATGCCGATGGGAGCGACACG
CTCACGGCGATTGCGAGCAAACTTGAAATCTATGCCGTGCGGTTCTGCCTTCTGCTCGCCCTCTCCGACTGGG
CGTGCGGGGCAAAAAAGAAGAAGGCGATAGAGGCGGCGACCGTTGAGAGGGCAATCCGTCTGACGGAGTATTT
CCGGGTAACGGCGACAAGGGTGCAGGGCATCATCGGCGAGGACGCGCTGACCGATGTGCAGCTTGCCGTGATG
TCGGAACTGCCCGCCACGTTCACCACTGCCGAGGGAGTTGCCATAGCCGCGAAATGCGGGATGGCGGAACGCA
CCTTCAAGAAATTCCTTCGCGACAGACGGGGCGTTTTCTTCACCCGTGATGCCTACGGCTCTTATTCCAAACT
TTGAATTTACCCCATGCACACCTTGCACTTTTGCACACTCCCGACTGTGCAAGAGTGCAAGGAATGCACGGGG
TTATCCCTGAATTTGAAATGATGACAACAGAGTTTTTTCTCGGTGTACCACTATACCCGTTACAGTGGTTTAC
CGCCCAGCGACCCATATCCGGGGAGCAGTCCCATCCGTGCTGCCGCGCACGGAAATTACAGCCATGCGGGAAA

GTCCTTAGCATATTAGGGAATTTTCCGAGCCGCATATCGTTCCGATACGCTGAAAATATCCCCAATATGCCAA
AGGGTGACCTCCCTTTGGAAACCCCGGAACGGCTTAACAGAAAGCCGTACACTCCGATTGCTCCGCAACTTAA
ACTCGTCCGCCTATGAGCTACGCAGTGTTCCACATCATCAAGGCGAACTCGTCGCTGAACTCGATAGCCAAGC
ATATCGAGCGCACCTCCCATCCCGAAAACGCTGACCCGGCGAGGACGCATCTCAACCGCTACAATCTGGTCGA
ATATCCCGACGGGATAACAGGGCTTGCGGCGGCTGTCGAACACCGCATAGCCAATGCAGGGATTACCCGGAAA
ATCTCCGACCGTCAAGTCCGCTGCCTAAACATGGTGATGACCTCCGACAACGAGGGTATGCAGAAAATCATCG
ACGAGGGGAAACTCGACGAATGGATTGCTGACAACATCAGATGGGCGCGCGACACATTCGGCGGCGATAATGT
TGTCGGAGCCGCACTGCACATGGACGAGCGCACTCCGCATCTTCACATCGCCGTCGTTCCGATAGTGACCGCC
GAGAGAAAGAAGAAAGCGAGGGAGGCGACCGCAAAGAAGCGTTACCGCACTAAAGCCGCCAACCGTCCGAGGC
TGTGCGCCGACGACATAATGGAGCGCGGAAAGATGAGCGGATATCAGGACACCTATGCCGAGGCTATGGCGAA
ATACGGTCTGGAGCGTGGCATCCGAGGCTCCGAGGCGCGCCACATCGGGCAGCACGAATATTACCGCGACTGC
ATGGTGAAGAAGAAAGGTCTTGAAGAAGACATCGGCAATCTCTCCACCGAGAAAAGAAAGCTCGACAAAGAGA
AAAAATCGCTGGAGAGCAAGGTTGAGAACCTTGAGTGCCGGACAACCGCCCTCGACACTCGGAAGAAAATGCT
CACGCAGGTCAACGAGGAGATACGGCAACAGAACAACGAACTCTATTCCGAAAACACCCGGTTGACCGAGGCT
AATTCTTCTCTCGCCACCGAAAACAAATCGCTCATGGCGACATCATCAAAACTCTCCGATGATGTTGTCCGGC
TGCAAATGCAGAAACTCTCCAACGAGGCAAACGGAGATTTCCAGAAATACAAGGCTGACATAGCCCGGTATTT
CCCTGACCTTGAAGATTTGCTGAATTGGGGGCGATACTGCACCCATATCGGATTTCCCGACAGATTCACGCAG
CGCATACTTGCCCAAGAAAAGGTAGGATTTGCAGGAGCCCTTTTCTCGTCGGAACACAATCAGACCTTTCGCA
CGGAAGGGTCGGTTGCCAAATTGGAAAGAGATCCGGAGAATAAACACCGTTTCATGTTGAATATCGACGGAGT
GAGCGTCTTTCAATGGTTCCGCAATATGGCTCGTAAACTCCTTGAGAAACTCGGCTTCAAACCAAAAGAGCCG
ACCCAAAGACACACCCGTAAAATTTAATTAACGTGAGTTCGACATAAGTAATCAGAACAACGTCAGTTGCTTG
TCAGCGACAAATTCCAAAGAGATAGACGGTTTCTTCGGGAAGAAACAATATGCAGCTATTGCACTCAGGGCAT
TGGTTATGAAATTTGCGAAAGACCGATGTCTTGAGTGTTCTATCTGAGCGATGTTCTTAAGTTCGTCATTAAC
AGATTCAACCAAAGCCCTTTTGCGAAGCATAATCTTGTCGGAAACAGACATCAGCGAGTTCCTCATGTTGTTC
TTGACTTTTGTGATAAGCTGAACTCCATTCATGAACAGAAACTCAAACAGTTGCTTGCCGATATATCCTTTAT
CTCCACAGAGCTTGCCCTTTACATTTTCAATAAATGACTCTGAATACAGAGGCTGACGGTCATCGACATTGCC
CGGTGTAAACATAAAGTTCAGAATCTCACCTTTGTCATTGATGATCAGGTGCAGCTTAAAGCCGAAGAACCAT
CCCATCGAGCATTTGCCCCGCTGGGCTATACCTTTGAAAGTCTTGTGTATAAGTATGCGTTGGTGTTTGCAAA
CTCTCAACGGTGTTGAATCAACATACGCAATGCCGGAACAAGTCCCAAGCAATACTTCTTTGATGAATACCGT
GAGTGACAACAGCACGGACTTTTGCAATTCAACAAATCTGTTGTAGGATACTGTCTTGGGGAACAAGTGCGTA
AGATGCTTGCAGACATACTGCATGTAAAAGTGTTTGAGGCATCGGAATCCCTTACTGTGAAAAAGAATCAGAA
TGGTAATGACCTCGGCATCGGAAAGCCGTCCCAGCTTATTGCGATGCTTCCTGCCATCACTGAGCTGATACTT
TTTGAGTTCAGAAGAAAAATACTTGCAGAAATCATCGGCAAGACAGAAAATTTCAGTAATTTTGTTCTCGGTA
ATCATAGCGCAAATTCTTGTAAATCTTGCAATTAGGCACTGTAAATTTACGGTTTTTTCGCGAGAATGCCAAG
AAAATCAGCCATTTAGTGTAAAACTAAATGGCTGATTGTTTTATTGGTGAATGGTCTATTGCTTATATCGAAC
TCACGTTAATTACTCAATAGCGACTATCATTGTTTTTATCAGTGATAGCCGCTATTATTTATAATTATTGACA
TAGTTCTACTCGGACACGGACGTTGGGCGGCGTTCGATTGAATGAAAATAAGCTGCTCCATAATCTTGTGGGT
TGTGGTGCCACAAAATTATGGAGCGAGTATACGGGTCGAAAAGACGCGGCTATTACTCTAAGCAGATAGCTTC
GCGTGTTAGCATAGATGGGTCGTTGCTTTCACAGCCTATTGCAATAAGTTCAAGTATGTCAGTTATTGCGCCC
TCTTTGTGAAAACCTCCTGTATGAATATATGCCCACTTTCCTTTTGCAAAAATAACCCAATTTACGCCATCAA
CACAAGGAATTTCTTTGAATGGTTTTGCCGTATCAACTATGTTTTGGAGAAATTGGGCTATGGCTGAAAAATC
GGCTGGATTTTCAATTTTCAGCTTGTATTCAGCATTTAATACCGGGGGCTGCGGTTCTTCTCCATTATCACGA
GCTTTATTGTATTCAATAAGCCAGGATAGTATGGGTTCCGAATAAATGCGATGAACAATACTATCACCATTGG
AGTAAAGACTAACGCCCATATCATAATCTTCAAGCCCGATAACCATAAAACCATAATCCGGCACACCGTCCAA
ATCCAATAGAAAATGTTTCTCTATTTGAAAGAGTTCGTTGTCAACATTTTCAAGCGGTTGCGCCTTTTGTTGT
GTGAAGCCAACAAAAGAGATTGTCATAGCGATGAAAAGTAGCGCGAGTTTCTTCATAATCGGAAAGAGTTTGA
GTTATTTGGAGAGAAGGACGCTTATTATTCAGTAGTTAACAAGTTCTAATCCTCCGAATACCGCAGTAACTGC
AAATATCCATGAAAATATTGAGGGTAAAATAGAAATATAGGAGGGAGCTTTACCGTATAATTTCATAGAGCCA
ATAAAGCCCAAAATGAACCATATAGAATATGAGTTTACAATAATCCACGCAATCGAGGCCAAATCAGGATGCT
TAACTCTATCCATAAAGAGAGAACCATAGAATACAAGAGGGGCTGCAATAATGGGAATGGCGAAAAGGAAAGC
TGCCACAAAAAACCATTTGGCTACATTATGTTTTTTCAAAAAATGTTTGCCGAAAATATATATACCGACTATT
GGCCCAAAGAAAGCTAAAGCCATAATGGTAATTAAAAATATAATGCTGATATTTGTCGTTTCCATACGCAAAG
TTACACAAAATCGGCGAGATACGGGGTATAATCAGAGGAAAACTTCGAGCGCAAAATCAAAACTTCCTCTCCC
AATCCTGCAATTCAGAGCTTCAAACCTATGGCAACTTAGAGAAAAAGAATCAAATAAAATATAACTAACGCTC
CTTAAAAATCCATAAACCCCTTTATTCTATTTCCTCTAATTATCGCAATAAGTACATTTTTAGTACACTTATA
TTATACTTATCTATTGATATTCAGCATTATAAATACTATACATCAAAACA
>MP_1_5_10_S46_clean_1_220871 flag=1 multi=3.0000 len=5096

GTATGTGGCGTTCAAGTCCAAGCGGGACAAGGATGACGAGGAACAACGGCTGGACAAGGCGCGGCGAACTGCC
GAGGTAACGCTGCGGGCATCCAAGGCCCAGATAGCCAAGATGGAGGCCGACGAGCTGCGGGGGAAGATGCACC
GCAGCGAGGATGTAGCCGCCATGACGGAGGATTTGATTTACACCATTCGTGGCTCGCTCATGGCCCTGCCCGG
GCGGTTGGCTGTGGACGTGGCTGCTGTGGCCACGCCCGCCGAGGCCGCTGAAATCATCCGTAAGGAAGTCCAC
GCCCTCATGCGGGAGCTGGCCAACTATCAGTATGACCCAGAGAAGTACGAGGAACGGGTCAGAGAGCGGAGAA
AGTGGGAGGCTGGCGGCGGTGATGTAGATGACGAGTAAATCCGCTACGCGGCGTTTGAACGGTGCAATCGCAA
AGGCCCTGGCCGGGATGAAGCCCCCGGACGATCTGACCGTGACCCAGTGGGCCGAGCGTCACCGGCGGCTGTC
CAGCGAAAGCAGCGCAGAGCCGGGACCCTGGCGAACCAGCCGGACACCGTACCTCCAGGAGCCAATGGACGCA
TTCACTGACCCGAAAGTGCGGCGAATTGTCATGGTGGCTGCGTCCCAGGTGGGAAAGTCGGAGCTGGAGCTGA
ACATCATCGGTTACATCATCGACGAGGATCCCGGCTCCATCTTGTTTGTCCACCCTACCACCATTGACGCAAA
GGAATTTTCCAAGCTCCGTATCGCGCCGGAAATCCGGGATTGCCCCACGCTGCGCCGGAAGGTGGCCGCGCCC
AAGAGCCGGGACAGCGGAAATACGCTGCTGCAAAAGACATACCCCGGCGGTATTCTGACCATGTGCGGCTCCA
CCGAGGCCCACGCCCTGGCATCCAAACCTATCCGCTATGTGCTGGGTGACGAGCGGGACCGCTGGGCCACATC
CGCCGGCAATGAGGGCGACCCCTGGGAGCTGGCCATGGCCCGCCAGACCACGTTCTATAATGCCAAGGCCGTG
GAGGTCAGTACCCCCACCATCAAGAATGCCAGTGCAATCGAGGCATCCTACGGAGAGGGTACGATGGAACGCT
GGAAATCCAGGTGCCCGCACTGTGGGCAGTACCACGAAATCCAGTGGGAGCATATTCGATACGACTACGAGGA
AAGCGTGGTCAATCACAAGAAGGTCTACAAGGTCAAAAGCGTGTGGTACAACTGCCCTGGCTGCGGCTGTATC
TCTGACGAGGCCACAATGAAGCGGGCTCCCGCCCGCTGGGAGGCGGAAAATCCCGCCGCCTATGCACAGGGCG
TCCGCTCGTTCTGGCTCAACGCCTTTGTCAGCCAGTGGGCGAGTTGGGCTTCTATCGTCCTCAAATATCTCAA
CGCCATAGGGAACACCCGGAAGCTCCAGGTGGTCTACAACACCTGTTTTGGGCTACTGTGGGAGGACCGCGGC
GACCTGGAGGACGAGGACAGTCTGATGGGGCGGCGCGATGTCGAGCCTTACGGCCTGGAAGAGGGCGGGACGC
CCATTGAGCTGCCTGACGGGGTGCTGGTGCTGACCGCTGGCGTGGACACCCAGGACAACCGCATGGAGTATGA
GGTCGTTGGCCACGGACACTTCGGGGAAACCTGGGGCATTGAAAAGGGGATCGTCATGGGGCGGCCCGATGAT
GACGAAACCTGGGCCAAGCTGGACGAGGTTCTGTTTAACCGGGTATTCCGCTTTGCGGATGGGCTGGGCCTGC
CGGTGTCGCTGACCTTTGTGGACCAGGGCGGCCACTTCTCCGGCGAGGTCCGGCTGGCGTGTCATGACCGAAT
CCGCAAAAAGGTGTTTGCGATTATGGGCTCCAAAAACCACGACGCACCTTATACGAAACAGCCCAAGGAACAA
AAAATCATGATTCAGGAGCAGTATCTCGGGACGTGCTGGATGTATGAAATCGGCGTCGACGCGGGGAAGCAGC
GGATTATGGAGAATCTGCGCGTTCAGACCCCCGGCCCCAACTACTGTCATTTCCCGCGCCGGGATGACTACGG
CTCCAGCTATTTCGCCGGTCTGCTGTCTGAGCGGCTGGAATATGACGCGAGCAAAAAGCAGCCCTGGGTATGG
AAGAAGATTCCCGGCCATGAGCGCAACGAGGCCCTGGACTGCCGCAACTACGCTATGGCCGCGTTCAAGGCTT
TGCCTAAAGACTTGGATGCCATAGACAGGCAGCTTAAAGCCATGCGGGGCCAACGCCCGCCAGCGGCTGTTGC
AACGCCACCAGCGGCCCCCGCAGCGCGTCGGCCCAAGGCTAAAAAGGCAGGCTCCAGACTGAACGAATATTTT
GACGATTGGTAGGTGGTATCATGGCAAGTGCTGTTGAGCTGAGGGCGCGGCTGGACTTCTGGCAAACCGCACT
GAAAAAGCTGCGCGAAGCATATCTCGCACTGGTGGATGGCGGCGTCAAATCGTACATGATCGATGACCGCCAG
CTGACCCGCTTTGATCTCCCTGCGCTCGAAAAGCAAATTGAGGAAGCGGAGGCCAAGGTGGACGAATTGGAAG
AGCTCTTGAATGGTCAGAGGCCCCGTCGGGCTTTTGGTGTGATCCCGAAAGATTGGTAACTGGGTAATTGCCC
GAAAGGGCTTTACCGAGGGTGGCCCGGCTGGGGTTTGTTCGCTCCTTTCGCCAGCTGGGCCACCCGCTTTTAT
AGCAGCATTGGAGGTGGACGCTATCTACTACAAAACCAAATCCGGCCTGCTGCTCCCTGACAATGCGCGGCCC
CAGGGCAAGGGATACAGCGAGGCCGGAGCCAGTCTGACCCGGCGGGCCATAAAAGGCTTCACGCCGCGCAGCG
GCTCACCGAACGAGGACATCAACTGGAACAACGCCACCCTGCGGCAGCGGGGGCGCATGCTCTATATGTCCGC
GCCGTTGGCCACGTCCGCCGTGAATACCAACCGAACCAAGGTTGTCGGTGTGGGCCTGACACTGAAAAGCGCC
GTCAACCGGGACTTGCTGGGCCTCTCCCCGGAGGCAGCCAAGGAGTGGCAGAAGCGGACGGAGGCCGAGTTCC
GGCTGTGGGCTGACCGAAAACAGAGCTGTGACGCTATCGGCATGAACAACTTTGACGGGCTGCAGCAGCTCTC
CCTTGTGTCGTGGCTTATGTCCGGCGATGTGTTCCCGGTGTTCAAACGATACCCTGCCACGCCTATCAGCCCC
TATTCCCTGCGGATCCATCTGGTGGAGGCCGACCGGGTGAGTACACCTGACCGAATGGGCGGCGGCGTGGGCT
GGCCGGGTATCACGGATGGGGAGAACTATCAGAACGGCAACAAGATTTTTGATGGGGTAGAGGTGGACAGGAA
CGGCATGGTCGTGGCCTACCACGTTTGCAACAACTACCCCTGGCAGATCACCGGCGACCCCAGCGTGTGGACG
CGGGTGGAGGCATACGGACCGCGTACCGGCCTACCCAACATCCTCCATGTGATGAACAGCGAGCGGTGCGACC
AATACCGGGGCGTCACCTATCTGGCCCAGGTAATTGAGCCGCTGCTGCAGCTGCGCCGCTATACAGAATCCGA
GCTGATGGCGGCACTCATTCAGAGCTTCTTTACTGCGTGGATCGTTACCGAGACAGACCCGAGCAAAATTCCG
CTCAACACGGTTGGCGCGAGCGGCATTGTTGGCATTCCTGGGGCAAATCCAGATGAAAACACCATCGCCGATG
ACCAGAATGAGTACGAGATGGGGCCGGGTATCGTCACGCATCTCAAACAGGGGGAGAAAATTGAGTTCGGAAA
CCCCAACATCCCCACCACGGGTTTTGATGTTTTTATCAAAACGTTCTGCAAGCTGATTGGCGCGGGCCTGGGG
ATCCCCTATGACGTGCTGATCAAAGAATACAATTCCTCCTATTCGTCCGCGCGGGCTGCGCTGCTGGACGCAT
GGGAGGATTTTCGTATGCGCCGGAAGTGGTTCGTGGACAATTTCTGCCAGCCCGCCTATGAGGTCTGGCTGGC
AGAAGCCGTGGCCCGTGGGCGCGTCAAGGCTCCGGGCTTCTTTGATGATCCCCTTATCCGCGCGGCATGGTGC
GGGGCGCGGTGGATCGGCCTACCCAGGGCAGCCTTGACCCACTGAAAGAGGCAAAGGCGGCAGTGCTGCAAA
TTCAGCACGCGCTTAAAACCCATGAGCAGGTCACCCGCGAGACGGGCGGCGGTGATTGGGATGAAAACGTGGA

GCAGGTGGCAGCGGAGAACGCGAAACTGGCTTCCGCTGGCGGCGGGGGTATCCGCATGGAAGTTGACCCCAAC
GAAAAAGACGATGACGAAGGAGATGGAGACAATGCCTAGCCCGTTGGAAATTATTTTCAACCGCAACCGCAAG
CCCAAGGCGATCAGTATCAACCGCGGCCCCTATGTCATGGAGATGGCAGACGGGGAAAATGCGGAGATCACCA
TGTACGGTGAGATCGTGGAAAGGCATCCGAAGCACTGGTGGACTGGCGAGCCGTTGGAGGGCGACTTCATCGT
GCAGGATGAATTTCTGCGCGACCTGGAAAACCTGTCCGGGGCCAAGACGCTGACCATCCGTATGCACAGCATT
GGCGGTGATGCCGGAGTATCTATCCTGATCCACAACCGGCTCCGGGAATTGTCGGCCAAGGGAACAGCCCTGA
CCTGTATCGTGGACGGCGTGGCTATGTCCGGCGGTTCGCTCATTATGTGCGCCTGCGATAATGTGGAGGTCAA
CCCCTCCAGCCTCGTTATGATCCACAAAGCAGCGATCTCGATTGAGGATAGCTGCAATGCGGATGAACTGCGG
AAACACGCTTCCGCTCTGGACGCATGGGATAAAGCCCAGGTGTCCATCTACAAGCGCAAGGCCCCGGGGCTTT
CCGATATGACGATCTCGAACATGATGGCAAAGACAACCTACATGACCGGGACGGAGGTTGTAGAAAAGGGCTT
TGCCAATAAGCTCCTGGAGGACGCGGAACCGCTGAACATTGCCGCCAGCGCCGACGGGCGCAGCCTGTTCGTG
CGCGGGCGGCAGGTCCACCTTATGCCTGGGGTATTCGCCCCGGACACGATCCCCACGGT
>MP_1_5_14_S33_clean_1_200524 flag=1 multi=22.9063 len=7753
TTGATGAAGGAGCCCCCTCATATTCCTATCTGATGCTCTTGTATTATCTTCATTCCTTTATCAGTTCCTTTCT
ACAGGCAATCAAAAACATTAAAACAAGCAATAAACCAATGACAGCATGAAACGGATTTTAATTATGACGGCAT
TTGGAAAAGGCGCGTGAGTTTGACCCATTCGGGCACGCGGGATTTGACCCCTCGGGCATAATAAGATTGACCC
CTTAAAAGTCCTGGCGGCGGGGGTCAATTTTATTTTACCTCAGTTGTTGTTTCAGGCCTTTTTCGCCTTGAGT
TTTCGCATACTTTCGCCTGTAAGCTCGATTGAGTGCGCTGAGTGGACTATCCGGTCGAGGATTGCGTCGGCCA
CCGTCGGGTCTCCCACCATGTCGTACCAGCTGGATGACGGATACTGTGAGGTGATTATGATGGATTTGCGCTC
GTGACGGTCCTCGATTATGTCGGGCAGGATCGGTCGCTCCTTGGAGTCGAGCCGGACAAGGGACAGGTCGTCG
AGTATAAGAAGCTGGCATCGTTCGATTTTCTTTAGCTCGGCCTCAAGACTGTTCTTGACCTTGGCCACCTTGA
GCAGCCCGAGAAGCTTGGCTGCGTTGGCGCAGAGTGTGCGTGTGCTTCTTCTGCAGGCCTCGTGCCCCAGGGC
ACAGGCAAGGAAGCTTTTGCCCGTGCCGGCAGATCCTGTTATGAATAGATTCTGTCCGTTGCGGATGAAGTCA
AGGGTAGCCAGACGTTCCATCTGGTTGCGGTCAAGACCGCGGCTGACCGTATAGTCGATTTCCTCGAGGTATG
CCTTGTAACGGAACGATGCGTTTTTTGTCAGTCTTGTCACCGCTGCCTGCGCGCGGTAGTCTCACTCGCGGCT
GAGCAGCATGGACAGGAAGGAGTCCGGGGTCATTGCCTGAGCAGTGGTTCCTGCCAGACTCTCGCGGAAGGCC
TCGGCCATTCCGTGCATTCATGCGGTTCATCAGATCCAGCGATATGGCGTTCTGGTCCGGCTGCTGGCTCACC
GGCGCTGTCTTGTCATTTGTTTTCATTGGAGTTGTCGTTTTTTACGTTTTCTTTGTGATTGACGCTGTAGTAT
TCGCGCCCCCGGATGTTTTTGTGCACAGGCGCGGGGGATGATGACACCGGGGTATGGACTGTCCCGTCCTCGT
CGGGAAGGAAGTCGGCGTCATCGCCCTGTTCAAGGACCTCACGCAGTGCCTGATATCCGTATTCGGATTTCTG
CGAGGCGCATACGCATCCGCCATGAGCCGGTCTGTCCCATATTTCTTCTCGAGCGACAGTATCCCCCGGCAGC
TGCTGAAGGATTTTGGCGGGTACTGCATCCGTCTGTCCACTTCCCGCAGGTAATCCAGAACGATATTGTCCAT
CTCCGCCGCACGTCGGAAGAGTTCCTCGAGGTCCTTGTCGTAAGGGCTGTAATGACCGGGCAGGTTGTGCTCC
TTCTTCCACGAGTATGTGTAAGGGATGTCGCATCTGTCGTGCGTCGCCACGAGGTTCAGACCGCAATATGTCT
CCACTGTGTCGGCATCGTAGAGGATGACCACACGCTTGCCCACATGTTCGCGGGGACGCTGCAGTGGTGCTTG
AACAGGGATACGTGGCAGTTCTTGCCGACTGTCATCAGTTTGCGTTCCTTCATGACAAACAGTTTCTCGGGCA
GGGGGCGGAGAAAGTCCTTCTCCCCGCGAAGGAAGATGTCACGGCGCGACAGTTCACGGCCGGCCATCGGCTT
CTCGTTGAAGTCTAGCAGCGATATGTGTATCGCCGTGTTCAGCTCATCCAGAGAGTTGAAAACCATTCCCTCG
ATATCAAGATAGACTGAACGGTACAGGAACTTGACCGCGTTCTCGACAAAAGCCTTGTCCTTGGGATGGCGCA
CACGCGCAGGGTATACGGCACATCCGTAGTGTTCGGCAAAGGCGGCGAACTCTTCGTTGATAACCGGTTCGTT
GCGGTCGCTGGTCTTCACCGCCGCCTTGAGATTGTCGGGAACGATGGCCGCCACAACACCCCCGAAGTACTGA
AAGGCGTTCTGACAGGCCTTGATCAGGTCTTCCTTGCGCTGGGACCATACGGCCTCGCAGTAAGTATAATGAC
TGAACGGAAGTATGGCCACGAACATCTCGGCCTTCTTGACCTCGCCCGTCATCTCGTCGACAACCGCGAGTCT
GTCGCCCGCGAAGTCGATGTACATCTGGTCGGCGGCGTAGTGCTCGACATGACCGACGACAGTGGTATGGAAT
CTGTATTGCCTGACGGCGCGTTTGAACACAGATTCCTGAAAGCCATCGGTATGACCTTCCAGATACTCATTGA
AGAGCTTACGCACGGTCGTTCCCTTGCGTGAAAGACGGGCGACATAATCCGGTATCAGCGCATCAAGTTCGAG
CTGACGCTGCGAAGGCTCGCGATGCCGGAACTCCGTGCAGCCGAACATCTCACGGAGCTGTTCCTCCGTAAGC
TCAAGCAGTCGCTCTGTCGGCTTGTCGCTTGAAATGAACAGCCGGACAAAGAGCGCATCTTCCGCTGCCCGCT
GTTCCAAATACATCTGTATCGTTCTCTTGAAATGGTCTGTCGTTTTCATATCGCTGTCGTTTTATCAGTTATA
CATTTTTCCGTTTCCCCAAAACTCGTAGTCGTTGGCTGCGCAACTCTCCTCGAAACTCTCCTCGCTGCACTGG
TATTCCATATCGTCACGGCACGACTTGAAGAAGCTGTCAAGGCACTTGTCCATGAGGTCGTACAGAGTGATTC
GGGTGTCGGGTGCTTTCAGAAAGTCGTAGATGGGTCGAAGAATGTCCTCGTCTGCGCAATAGCCTGTCAGTAC
GCAGCAATTGTCCGTGAATACACTGCTTTTGCGTCTTTTTTTGTAATTGCCTTTGAGGTAGTAGGTTCTCGGC
TTGAATAGGGTGTGCCAATAATTATTGACGATGTATTTCAGAAGCCGTATGCCGCTGAGTTCCTCTTCCTCTC
CGCTGTAACGTGAGGTGAAGCGGTAGGAATAACGGCACGTGTCGTAACTCCAATCGTTCACTTTGACTTTGAA
CACACTTTCAAATGCTTCCAGCGTGTTGCGGTTATCGCTGTCCCAACCGTATTCAAAGGTGAGTAACCATTCG
TTGTATGCGGTATCTCTCGCTGCTGCGGAGAGTTCGGCTAATTGGTAAAGGTCGTATGTCCTTGTTATCGTTC
TCATATCGCTGTCATTTTAGATGGTCTGTTTCAGTATCTCTCTCCAATAGGTCGGCTCCACCTCGCTCAAAAG

AAAGTCCGTAAAGTCCCTCCGTGCGTCCTTGTTCAGTTCTCGGTAGAGTTCACAGAACACGGATATGTTGCCG
TTTATGTACGTTTCCACCATGTACACGAAGATGTTGTCCACCTCGTAGTATCTGCATTGCTGCGCTGCCGTTT
TGCTGCTTCTCTTTGCCATGTCCGTAGGGGTTAAAGGGTGAATAACCAAAGGATAAAGCCGAAGAAGGCGATA
GTGGAAAGGATAGCCACGATTACGCCTATTGCCACTCGGAACACTCCGTTTACAATCTCTGCTATGATGCCCC
AAAGGATGCCGAACACCCCGAAGGCGGTGCGTATTATCAAGCCGCATATCGCCAACCCGATGTATTGCGCAGT
TTGTCTGAAATTTGCCGTTGCCGTCATATCTTCGCTGTTTTTGATTTTTTAATGCGGATTCAAGAGCTGAGG
GAGTTGAGTTTCAAACTATCTTATCTGCCTCTCGTTTATCCGACATTTTTTAATGCGTCTTTCTGTCGCATC
GGTCGTTTTCGTTTCGGGTGCTTGAAAAGGTAGGGATTAGGGGATGCAAGGTTGGCTGGAGATTTTTGCCGAA
AACAGGAGGTTTGACCTTGCATTCCCGTCCAATCCCGGAATTACCTTTGCGCCCAGAACGGAAATGACTGCCT
GATGCGGCTCACTGAAAGGCGCGGCAATGGAGGTAAACGGAAGTGGAGGCAGATTGGACAGGAAAACTTCAAA
AGAGAAATCCGTAAAAAAGGGATAATGCCAAAAAGAAAAGGGACACTGCCGGAAGCGTGAAACCGGGCAAACC
ACCGGCAAGGAAGTATGATTTTATATGTCTGGCCGAGCGTGTCCGGTCTGACGGATAAAATCATTCTTCCCGG
TTTGCCTGCCGTGTATGACGGCTTGTTCCATTTACGGATCGGGCGGTCATGCGCGGCTTTCCGCTTACGGCTC
AAAGGAACAGCAAAAAAGAAAAATCGTCAGAAACCCGTAAAAGCACCTCTCTGGCATAAGCGCAAAAGAAATG
GGCCTTGCATCATCAGTCTAAACTTCTGTTTAGGCGTGAGGCAAAGCCCTTTTCTCCGCTTATGCGGTAGTCG
GCATACGTTCGTCCAAAATCTCTTTGTGCGATGGTGTGTCGATGGAGAGAACCGCTTTTACGGAAGATATGAA
TACGAGTATATCAAAAAATCACACTCGAAACACCGAAAAAAAGTAAATCATTGGGGAGTTTGGATAAAAAAGT
GTACCTTTGCAATAGAGTTGTTAGATGTAGCAAAGCACAGCATACCAGTGAATTTGCATTGTCGCTAAATCGT
TACCATAAAATTGAAAATCTCTTATTCTATTCTGACACACAATTAGATACAGTTTTTTTGACTATCCGGGGCA
AAGAACCTCCAGAAGCTCGGACGTACTCAGGAAGCACTCTCCATGATGGAAAAATATGCCATTGCCCGCGACA
GCATCCGTGAATCCAAAAACAAACGAGACATCAATTCCTTTAAGGTTAAATTTGAAACGGAGAAGAAAGAACT
CCTTATATCCAAACAGAAAGGTGAACTCCGGATGCGGGCCATTCTTCTCTGCCTTGTCGTATTTATACTCATA
GCATCAACTTGCGGATTCATAATATATATAAGGAACCGTAACCGCCTCTTCCGTCTCATAGTGGAACAGCAAA
AAGAATTCACAACCAGACAAAATAAATATGCCCCTTATCTCGACAAGAATCAGGAGATCAAGGAAGGTAAATA
TGAGGAATATGATAAGGATTCACAAAACTATACCAACTATATAAAAGATAAAAGTTCTGGAAACGGATTGCCG
TCGCAAAAAAAGGCCGATGCCATCTGGCGCGCTATTCTTTTAGCGATGGAGACAAACAGGATATATACCGACC
CGAATGTGACCCGAGACTCTTTTGCTGAAAGAATCGGCACGAACCACACATGGCTCACTGCCATTATAAAGGA
TCGTACAGGCAAGTCATATACGCAATTCATCAACTCCTGGAGAATAAACGAAGCAGTCAAAATTCTGTCGGAA
GAAAACTGTGGCTACACAAACAAGGAGCTGTCGGAACTGCTGGGATTCATGACTCCTCAATCGTTCTACAACA
CTTTCCGTCAGCAAATGGGAATGTCGCCTGCCCGTTTCCGTCAAGACATCCTCTCCGTATCCACAGCAGATAA
AACAGAAGATTAGCAGAATGAATAACCGGATTATTGTAAAATATCCGCAAATTCTATAATGATTCAGCCATTT
GCGATTTGCTTACGCGCTGATACTTTGAACATTTGATTTTCCATCATACTTTTGCTGCCACAGTTCAGGCACG
TTGCCTGACTGAAAAACAGCAATACTCATGAAAACTTTTTACAAAGGATTGATCGGGGTTACGGCATTCGTAG
CAGTGTCGGCAGCTATCCCCGCAATGTCGTCCGCACTCAGCCGCGACCGCCTCGTCAAGGCAGACCGCCTCCA
TCAGGAGAGCGAGATTTCCACTCCCGGACAAGTAGCACAACGTGCTCATAAAGCCAACAGGACCGGCACACCC
TATTGGCAGACGGCCAAAAGCATCTACAACGCCGATGGAAAGACAGACAAAGAGAACGGACTGACAAGAGAAT
TCGGTACGCATATCGAAATCGATGGCGAGACCGCGAGAATCTACGGACTCGTAGATGTCAACTACAGCAACTA
CTACGAAATCGATGAGGAATTCGCCGTAGAGGGAGTATATAACGAGCGCTACGGAACAATCACAATCAGCGGC
ACAGACTACAACCCGGAACGCCCTCTGTCGGAGTACAACAGACTAGCAAACATCTACTCTCCGGCGGATGATA
TGGCATATACTGTCGTGCTGATTGCAGGAGACTGTAATGAAAGAGGTGAAATCAATACAAGCGAAGAGCTTAT
ATTCGATGTTTCTGATGATTTGTCGACAGTACGCCCCAAGAAGGCTTACGGAGCATACGCTTTCACCTCCGAA
GGTGAAGGGAAAGCCTTTGTGGACTTCTATCAGTCTTCCAGCATACTAAAAGCGCCGGAAGAGCCAGAGCTTA
AGACAAATGCTGAAACCATTACATTCAAACGCCAGTTCGTAGCGGCAGGAATGCCGGTCATAGAACAGTTCAA
CCTTATGAATGCTGGCGCCTCGGATTGCACGTTTACAGTTTCCACATCTTCTCCTTTGCTAAAAGTATCACCG
GAAAAAAATTCCCTGGAAGGTTGTTCCTCTGTGCCTCTCACAGTCACATTTTCACCGGAAGAGCCAGGTATCT
TTGACGGAAAGATAACTATAAAAGGAGCCGGTAAAACAATTGAAGTGTCTGTAAACACCGAGGTAAGAGAGAT
CCCAGACTACACACGTATCGTTAAAAGCGGAAGCAATAAAATTGAATTCGAGAGGTCTCCGGTCTACCCTTTC
GTGCTAAGCGATGAAAATGGCATTACAGTGGCGAAATCCATCAACAATGGCGAAGGGAATGACACCGAGTCTT
GGTTCAAATGTATTGTTGATGTTCCGGATGGACAATCAGGGTTATTCTCTTGGAAAGCTGTTCAGGAGGACCG
CCAGCCGAATACTCTCGCCATACTACTTGACAATGAATTGGTTAAAAGCGAGATATATCGACCCTACGGGCGG
AGTAAGCACCTGCACGGAAGCAGGTACGGAGAAATACGAAAAATTCCAAATGGGTATCGGCAGACGCAAGCGG
ACACTTGTGCAGTACGACTACCGCCACCCCGCAGACGGAGAGTTGTTCTCTTGTGTCAAACCCACGTTGGACG
AGTGCCGGACTGCACGTGACAAGTGGCTCACGGCGAAGGAAAGAAAGGAGGACAAGCGATGAACGCAGTAATC
TACCAAACGCTGATAGTGAAGTTCAGCGAGCCTATCAAAGTATTGGATAGCATCTTCGACGATGCGGAAGCGT
GGGGAACGGACACGCTGAAAGGGTGGATAGACAACTACGAGGGAAGCAGGTTTACCGCCATTGACAGCCATAC
GGCTGTAATCACAAGCGAGTACAACATGGAGTGTCTGAAAGCGTGGCTGGAAAGGCACACCCCCATAAACGAG
AAAGCAGAATTTTAAGCGTGTTGGCGGTGTACGCACCGCCAACATATAAACCCTAAAAGACAGCGTATATGAT
AGCGAAAACAATTTTGGAGCAGATAGGCGGCAGACGCTTTGCCGCCATGACTGGAAGCAAGGACTTCATAGAC

ATGGGCAACGGCTTACGCATGAGCCTTGCGAGGAACAAGACGAGCGCAAACCGCCTTGACATCATCTACGATG
CAGGATTAGACCTCTACAATATGCGTTTCTACCGCAGGACGTTCAGCAAAAAGACATTCGAGTGCAAGACGAA
GGACATCGAAACGCACAAGGGGATATATTGCGATATGCTGGAAGAAATGTTCACGATGGTAACGGGGCTTTAC
ACCCGATTTTGAGGG
