# Supplementary material for: Fecal DNA Virome Is Associated with the Development of Colorectal Neoplasia in a Murine Model of Colorectal Cancer
Source: Pathogens. 2022 Apr 11;11(4):457. doi: 10.3390/pathogens11040457 (PMC9025118; doi:10.3390/pathogens11040457)
Supplement: Supplementary file 1 [file pathogens-11-00457-s001.zip › Supplementary Figures S1-S4.pdf]

Supplementary Figures

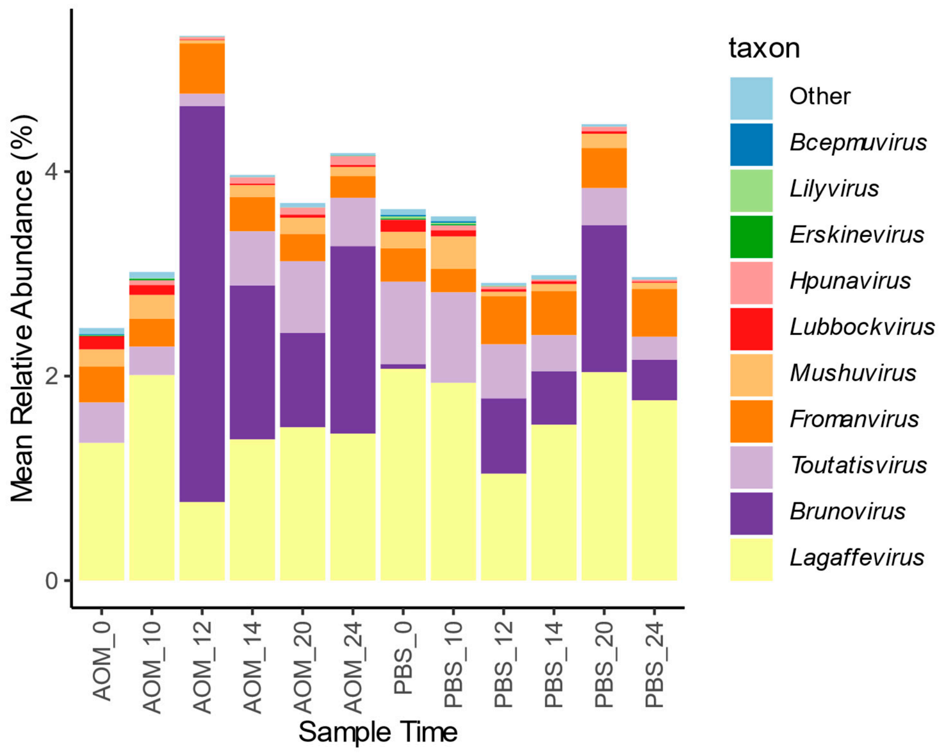

Supplementary Figure S1. Stacked bar plot of classified viral genera with top 10 mean relative abundance (%), other viral genera were grouped into other.

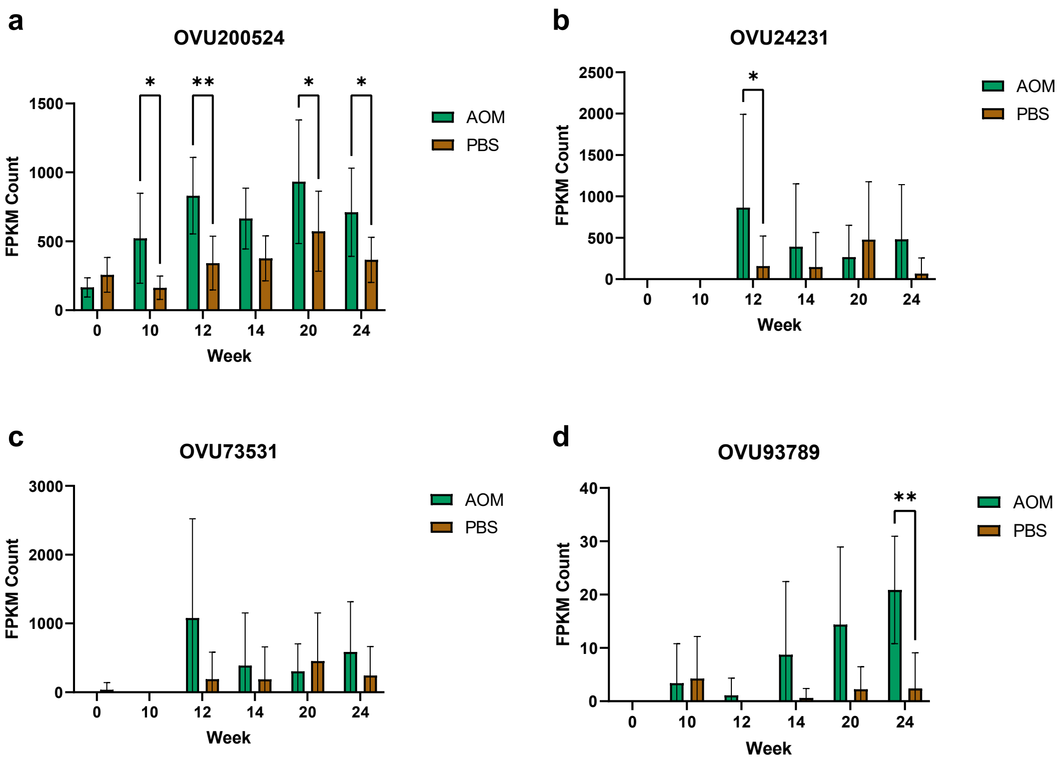

Supplementary Figure S2. Mean abundance (FPKM count) plots of OVUs that are significantly correlated with tumour size and the number of tumours. a) represents OVU#200524, b) represents OVU#24231, c) represents OVU#73531, d) represents OVU#93789 (*P≤0.05;**P≤0.01).

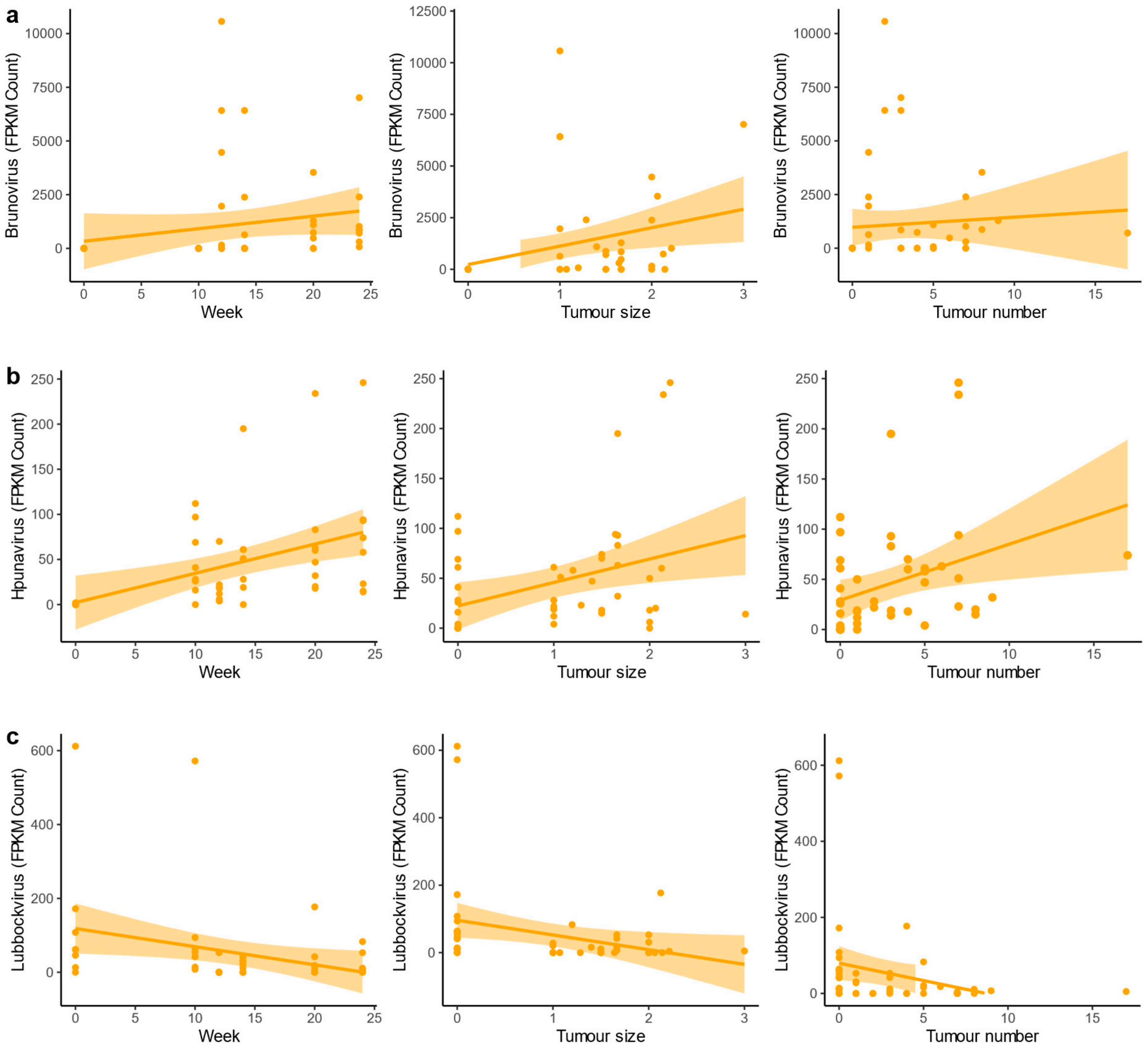

Supplementary Figure S3. Scatterplot of the abundance (FPKM count) of important genera vs week, tumour size and tumour number. a) corresponds to *Brunovirus*, b) corresponds to *Hpunavirus*, c) corresponds to *Lubbockvirus*.

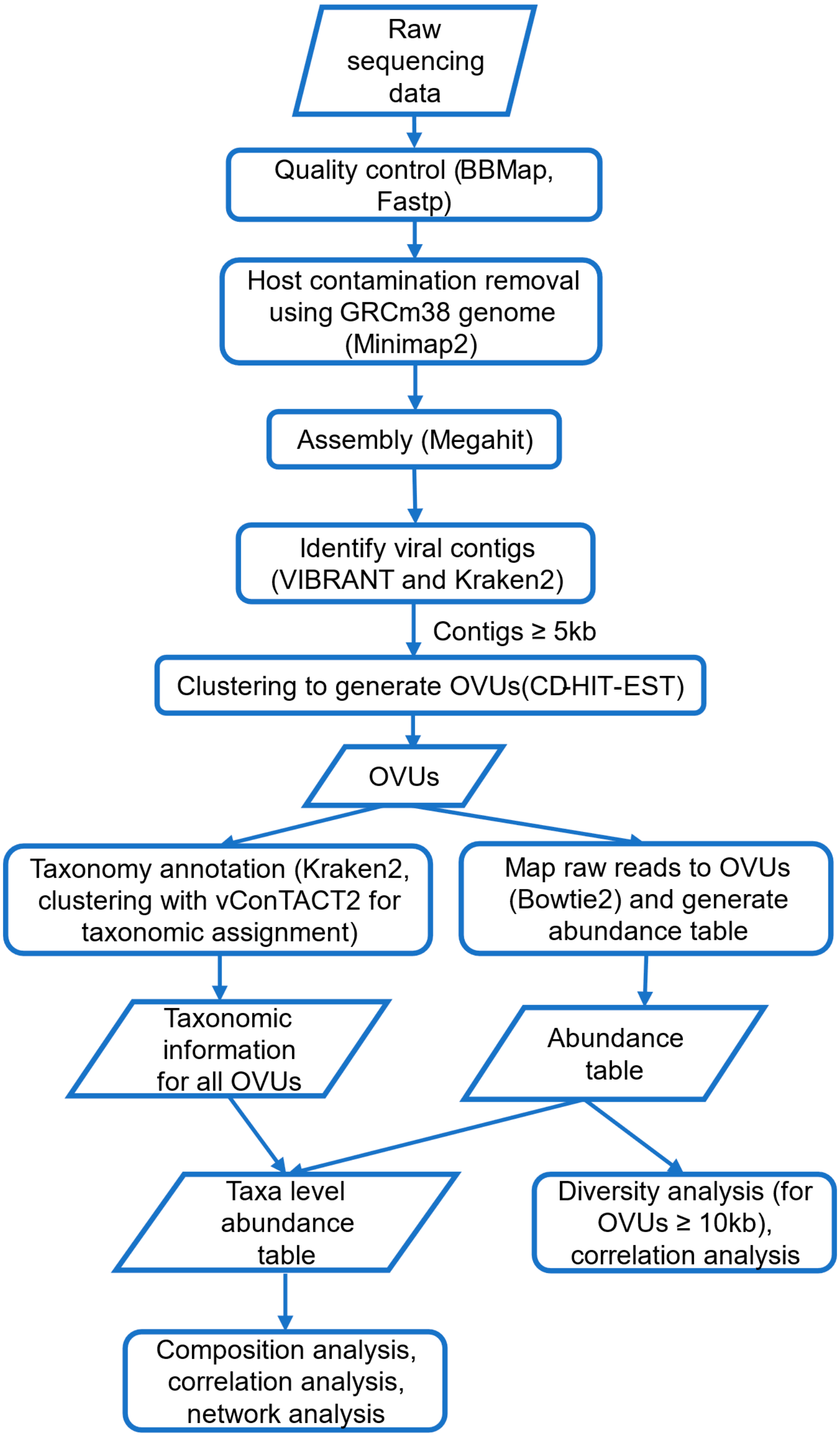

Supplementary Figure S4. Bioinformatics pipeline for analysis of virome sequencing data. The raw sequencing data were first subjected to quality control and host contamination removal. The clean reads were assembled, contigs that belong to viruses were identified and clustered to generation operational viral units (OVUs). Raw reads were mapped to these OVUs to generate an abundance table for diversity analysis and correlation analysis at the OVU level. These OVUs were also taxonomically annotated for taxa level analysis.

Supplementary Data

Supplementary Data S1. AOM PBS mice characteristics

Supplementary Data S2. Comparisons of the observed number of OVUs and Shannon diversity in different sample times. P was calculated by pairwise Wilcoxon test, Q value was adjusted by Benjamini–Hochberg method. Sheet a corresponds to the observed number of OVUs, sheet b corresponds to Shannon diversity.

Supplementary Data S3. Comparisons of beta diversity in different sample times. *P* value was calculated using pairwise.adonis from the R package 'vegan'. Only p.adjusted values ≤ 0.1 are considered significant

Supplementary Data S4. Full differential abundance table at OVU level. Ordered by week and AOM logarithmic discriminant analysis (LDA) score reported by linear discriminant analysis effect size

Supplementary Data S5. Correlation analysis between the abundance of OVUs, week and tumour characteristics. *Q* value cut-off is 0.05

Supplementary Data S6. Fasta sequence of OVUs that are significantly correlated with week, tumour size or tumour number

Supplementary Data S7. Correlation among viral genera and between viral genera and tumour characteristics. Normalised local similarity (LS) value was calculated by dividing all LS scores by the absolute value of the maximum LS score
